# Germ granule dysfunction is a hallmark and mirror of Piwi mutant sterility

**DOI:** 10.1038/s41467-021-21635-0

**Published:** 2021-03-03

**Authors:** Maya Spichal, Bree Heestand, Katherine Kretovich Billmyre, Stephen Frenk, Craig C. Mello, Shawn Ahmed

**Affiliations:** 1 Department of Genetics, University of North Carolina, Chapel Hill, NC USA; 2 Department of Biology, University of North Carolina, Chapel Hill, NC USA; 3 RNA Therapeutics Institute, University of Massachusetts Medical School, Worcester, MA USA; 4 Howard Hughes Medical Institute, Worcester, MA USA; 5 Lineberger Comprehensive Cancer Center, University of North Carolina, Chapel Hill, NC USA; 6 Present Address: Stowers Institute for Medical Research, Kansas City, MO USA; 7 Present Address: Achilles Therapeutics Limited, London, UK

**Keywords:** Epigenetic memory, Germline development, Gene regulation, Piwi RNAs

## Abstract

In several species, Piwi/piRNA genome silencing defects cause immediate sterility that correlates with transposon expression and transposon-induced genomic instability. In *C. elegans*, mutations in the Piwi-related gene (*prg-1*) and other piRNA deficient mutants cause a transgenerational decline in fertility over a period of several generations. Here we show that the sterility of late generation piRNA mutants correlates poorly with increases in DNA damage signaling. Instead, sterile individuals consistently exhibit altered perinuclear germ granules. We show that disruption of germ granules does not activate transposon expression but induces multiple phenotypes found in sterile *prg-1* pathway mutants. Furthermore, loss of the germ granule component *pgl-1* enhances *prg-1* mutant infertility. Environmental restoration of germ granule function for sterile *pgl-1* mutants restores their fertility. We propose that Piwi mutant sterility is a reproductive arrest phenotype that is characterized by perturbed germ granule structure and is phenocopied by germ granule dysfunction, independent of genomic instability.

## Abstract (web summary)

Piwi deficiency results in sterility and is associated with transposon expression and genomic instability. Here the authors show that sterility of *C. elegans* Piwi *prg-1* mutant is not associated with transposon-induced DNA damage but is associated with and is phenocopied by dysfunction of germ granules.

## Introduction

Germ cells give rise to the mortal somatic tissues while maintaining themselves in a pristine condition that allows them to be propagated for an indefinite number of generations. When defects in pathways that promote “germ cell immortality” are compromised in *Caenorhabditis elegans* (*C. elegans*), this results in a Mortal Germline (Mrt) phenotype, where robust fertility occurs for several generations but then deteriorates and finally culminates in sterility^1,2^. The germ cells of *mrt* mutants may transmit a form of damage that accumulates over generations and ultimately evokes sterility. Understanding the causes and consequences of transgenerational sterility in *mrt* mutants may provide insight into the unique biology of germ cells.

The initial germ cell immortality pathway defined in *C. elegans*, the telomerase pathway, maintains telomere length by adding de novo telomere repeats to chromosome termini^3^. *C. elegans* mutants that are defective for telomerase display progressive telomere erosion, which ultimately triggers telomere uncapping and end-to-end chromosome fusion^4,5^. This could lead to the formation of circular chromosomes that are toxic for meiosis and might evoke sterility^6^.

Telomerase is suppressed in somatic cells of humans and other large mammals, which results in telomere erosion. This serves as a “biological clock” that suppresses tumor formation but promotes aging by triggering a form of permanent cell cycle arrest termed senescence^7–12^. Somatic cells of short-lived mice possess telomerase, but accumulate other forms of stress that increase transcription at the p16^Ink4a^ locus, which can induce senescence independently of shortened telomeres. Aside from telomere erosion, forms of stress that accumulate to cause senescence in mammals are not well understood. We therefore reasoned that *C. elegans mortal germline* mutants that are not defective for telomerase could uncover additional pathways or factors that regulate cellular senescence and aging. In this regard, deficiency for the *C. elegans* Argonaute protein Piwi/PRG-1 and related proteins that promote small RNA-mediated genome silencing compromises germ cell immortality^8–11^ but does not result in creation of end-to-end chromosome fusions that are a hallmark of late-generation *C. elegans* telomerase mutants^4,5^.

Piwi is a conserved Argonaute protein that interacts with thousands of piRNAs in germ cells and represses expression of transposons and parasitic nucleic acids that represent threats to genome integrity^8,12–14^. P-M Hybrid Dysgenesis is a temperature-sensitive *Drosophila* fertility defect that is associated with expression and transposition of a subset of transposons, including the P-element, which are normally silenced by Piwi/piRNAs^15,16^. Deficiency for Piwi proteins leads to immediate sterility in *Drosophila*, zebrafish, and mouse, and correlates with increased transposon expression and DNA damage in homozygous mutant animals^14,15,17^. However, analysis of *Drosophila* strains with varying levels of transposons suggests that transposon-induced genome instability may not convincingly explain the infertility associated with P-M Hybrid Dysgenesis^16^. Therefore, the cause of sterility in Piwi mutants remains an open scientific question.

Deficiency for the *C. elegans* Piwi ortholog *prg-1* does not induce the immediate sterility phenotype observed in Piwi mutants from other species, but instead compromises germ cell immortality^8^. The delayed sterility of late-generation *C. elegans* Piwi pathway mutants^8,9,11^ could result from gradual decay of Piwi/piRNA-dependent histone silencing marks as germ cells proliferate over several generations. Heterochromatin dysfunction in *C. elegans* has been associated with increased levels of transposon and tandem repeat expression, with RNA–DNA hybrid formation, and with transposon-induced DNA damage^18–20^. However, *C. elegans* mutants that display high levels of transposition do not become sterile unless grown at elevated temperatures, so the relatively low levels of transposition observed in late-generation *prg-1*/Piwi mutants are unlikely to trigger *prg-1* mutant sterility^8^.

Sterile late-generation *C. elegans* Piwi mutants display a pleiotropic germ cell degeneration phenotype as L4 larvae mature into 1-day-old adults, such that most sterile adults possess germlines with only a small population of mitotic germ cells (atrophy), whereas some germlines lack germ cells (empty), some germlines possess both mitotic and meiotic germ cells but are under proliferated (short), and some germlines are no smaller than germlines of fertile late-generation *prg-1* mutant siblings (normal)^21^. Atrophied germlines of sterile *prg-1* mutants can regrow on day 2 of adulthood, and fertility of a minor fraction of sterile late-generation *prg-1* mutants can be restored by altering their food source^21^. This implies that Piwi mutant sterility is a form of reproductive arrest. It has been previously demonstrated that severe environmental stresses like starvation can induce states of reproductive arrest that have been termed Adult Reproductive Diapause and Reproductive Quiescence^22–24^.

Germ granules are electron-dense perinuclear foci found in germ cells^25^. *C. elegans* germ granules were originally identified by antibodies that label P granules, which mark the germ cell lineage^26,27^. Mutator bodies and Z granules are substructures within *C. elegans* germ granules that lie next to and intermix with P granules to promote small RNA biogenesis^28–31^.

Here we demonstrate that germ granule abnormalities typically occur in sterile but not fertile late-generation *C. elegans* Piwi pathway mutants. Dysfunction of genes that encode germ granule components can enhance the infertility phenotype of *prg-1* mutants. Moreover, germ granule defects can mimic infertility phenotypes observed in late-generation *prg-1*/Piwi mutants. Together, these findings show that P granule dysfunction not only correlates with but is sufficient to mimic a form of reversible reproductive arrest that is characteristic of sterile Piwi mutants.

## Results

### Genome instability in Piwi pathway genome silencing mutants

Some Piwi pathway-mediated genome silencing mutants only become sterile when grown at the non-permissive temperature 25 °C^10,11,18,32–34^. However, animals lacking the Piwi Argonaute protein PRG-1 or the nuclear RNA interference (RNAi) proteins NRDE-1 or NRDE-4 develop progressive sterility at any temperature^8,18,32,33,35,36^.

**Fig. 1:**
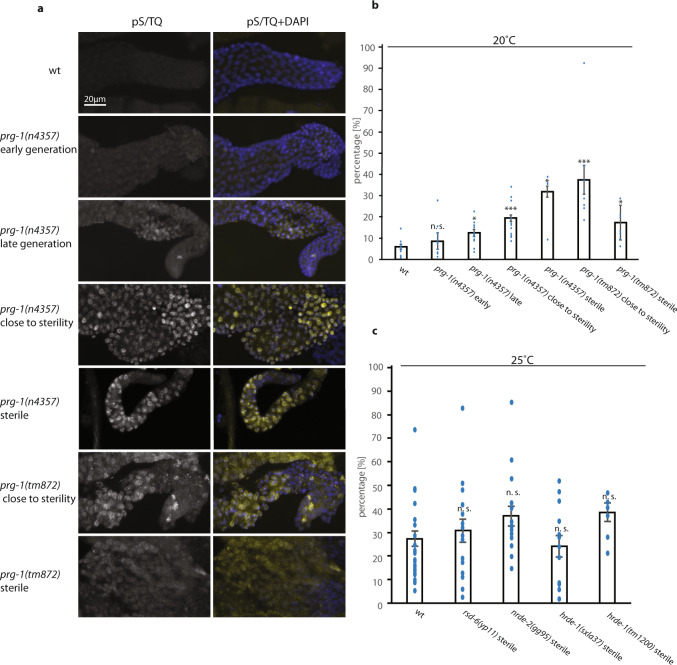
DNA damage response is variable in Piwi pathway genome silencing mutants prior to and at sterility. **a** Staining of pS/TQ in wild type, *prg-1(n4357), and prg-1(tm876)* mutants. pS/TQ (gray and yellow) and DAPI (blue). pS/TQ staining in wild type, *prg-1(n4357),* and *prg-1(tm876)* mutants was repeated three times with similar results. Representative images are shown. **b** Quantification of pS/TQ staining in 20 °C wild-type and *prg-1* mutants at different levels of fertility (wt *n* = 14; *prg-1(n4357)*: early generation *n* = 14, late generation *n* = 12, close to sterility *n* = 11, sterile *n* = 4; *prg-1(tm872)*: close to sterility *n* = 8, sterile *n* = 8. Error bars represent the standard error of the mean (SEM). *p*-Values were obtained by comparing sample groups in a two-sided Mann–Whitney *U* test with Bonferroni correction (*p* >0.05 = n. s., *p* <0.05 = *, *p* = <0.01 = **, *p* = <0.001 = *** compared to wild type). **c** Quantification of pS/TQ staining in 25 °C wild-type and small RNA genomic silencing mutants at sterility; wt *n* = 25; *rsd-6(yp11)* *n* = 17; *nrde-2(gg95)* *n* = 17; *hrde-1(sxla37)* *n* = 13; *hrde-1(tm1200)* *n* = 6. Error bars represent the standard error of the mean (SEM). *p*-Values were obtained by comparing sample groups in a two-sided Mann–Whitney *U* test with Bonferroni correction (*p* > 0.05 = n. s., *p* < 0.05 = *, *p* = < 0.01 = **, *p* = < 0.001 = *** compared to wild type).

We asked if DNA damage signaling is directly associated with Piwi pathway mutant sterility, a topic that has not been previously studied. We studied the DNA damage response using an antibody to phosphorylated S/TQ, a protein modification that is created by two sensors of DNA damage, ATM and ATR, which are phosphatadylisonisol-3-kinase like protein kinases^37,38^. We examined early- and late-generation *prg-1* mutants grown at 20 °C and observed a significant increase in the fraction of germ cells with upregulated DNA damage response for late-generation fertile and sterile *prg-1* mutants (~3- to 6-fold) when compared to either wild-type controls or to early-generation *prg-1* mutants (Fig. 1a, b). However, comparison of two different alleles of *prg-1*, *n4357* and *tm872*, revealed that the fraction of germ cells with an activated DNA damage response was not consistently elevated in sterile *prg-1*/Piwi mutants in comparison to fertile late-generation siblings (Fig. 1b). We also tested temperature-sensitive Piwi pathway mutants and found that the wild-type DNA damage response was upregulated by growth at 25 °C but was not further elevated in sterile late-generation *rsd-6, nrde-2* or *hrde-1* mutant germlines (Fig. 1b, c). Together, these data suggest that the sterility of Piwi pathway mutants is not consistently associated with elevated levels of DNA damage signaling.

### Piwi pathway genome silencing mutants exhibit germ granule defects at sterility

**Fig. 2:**
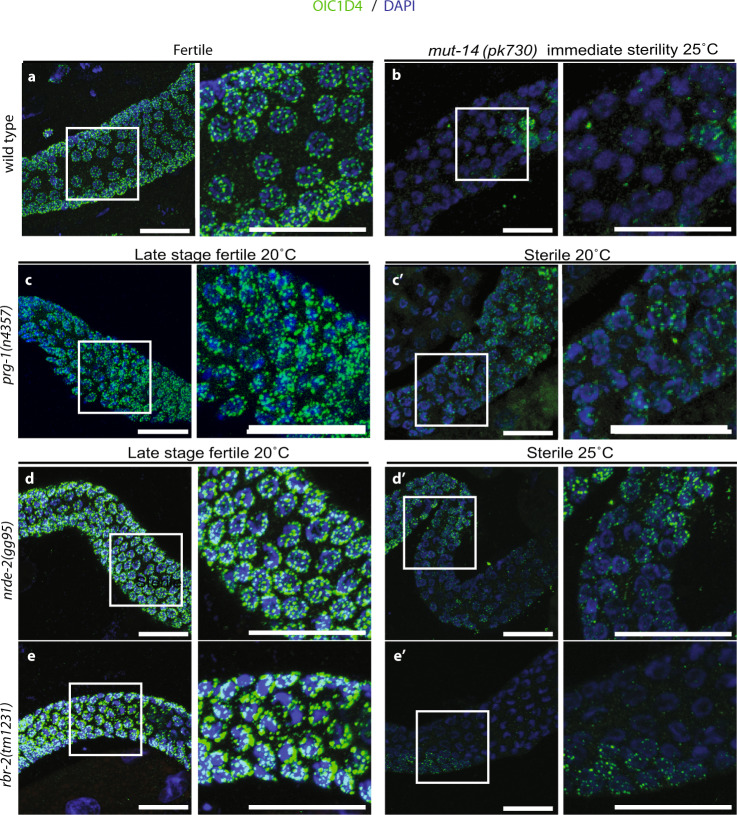
At sterility, partial loss of P granule staining occurred in *prg-1* and temperature-sensitive mutants. Germlines of sterile days 2–3 adult animals were stained using the OIC1D4 antibody against PGL-1 in P granules (green) and DAPI (blue). More than five independent OIC1D4 antibody immunofluorescence experiments were performed on wild-type and different mutants with similar results and at least five animals were scored for each condition (Table S1). Representative images are shown. Scale bars indicate 20 µm. **a** Control animals contain uniform puncta of P granule staining surrounding each nucleus; **b** *mut-14* mutants display an immediate sterility when shifted to 25 °C for one generation and display P granule abnormalities. **c**–**e** Late generation fertile *prg-1, nrde-2*, and *rbr-2* animals displayed relatively normal P granule staining in the germlines; **c**ʹ–**e**ʹ *prg-1*, *nrde-2*, and *rbr-2* sterile siblings all exhibited some degree of germ cell P granule loss.

We initially asked if the expression or localization of P granules correlates with sterility of late-generation Piwi pathway mutants by staining germlines of fertile and sterile late-generation adult Piwi pathway mutants with an antibody (OIC1D4) that recognizes either the P granule protein PGL-1 or a protein that is required for PGL-1 stability or localization^26^. We found that perinuclear PGL-1 staining was frequently strongly reduced or absent in sterile late-generation *prg-1*, *nrde-1*, and *nrde-4* mutant germ cells, but not in late-generation fertile siblings (Figs. 2c, cʹ, S1, and Table S1). Similarly, growth of the temperature-sensitive Mrt mutants *nrde-2*, *rbr-2, rsd-6*, and *hrde-1* at the restrictive temperature of 25 °C resulted in sterile late-generation animals with marked reduction in PGL-1 staining, which was not observed in fertile late-generation siblings (Fig. 2d, dʹ, e, eʹ, S1, and Table S1).

Many but not all sterile Piwi pathway mutant animals experience germline atrophy as L4 larvae develop into adults^21^. We therefore asked if germ granule defects occur at the L4 stage of development, prior to the onset of germline atrophy. We observed P granule dysfunction (as measured by lack of PGL-1 staining) in some L4 and day 1 germlines of *prg-1* (L4: 28.57% *n* = 7; day 1: 50% *n* = 10) and *nrde-4* mutant animals (L4:60% *n* = 5; day 1:50% *n* = 4) (Table S2). Thus, P granule loss already appears in the developing germline prior to germline atrophy (Fig. S2).

Apoptosis contributes to but is not essential for this large-scale germ cell degeneration phenotype of sterile *C. elegans* Piwi pathway mutants^9,21^. P granule proteins PGL-1 and PGL-3 have been shown to vanish in wild-type germ cells that undergo apoptosis^39,40^. However, some sterile late-generation *nrde-2* and *rsd-6* mutant adults had large wild-type germlines despite displaying strong depletion of PGL-1 expression (Figs. 2d, dʹ and Fig. S1f, fʹ). Therefore, depletion of P granule proteins in sterile Piwi pathway mutants is not simply a consequence of apoptosis.

**Fig. 3:**
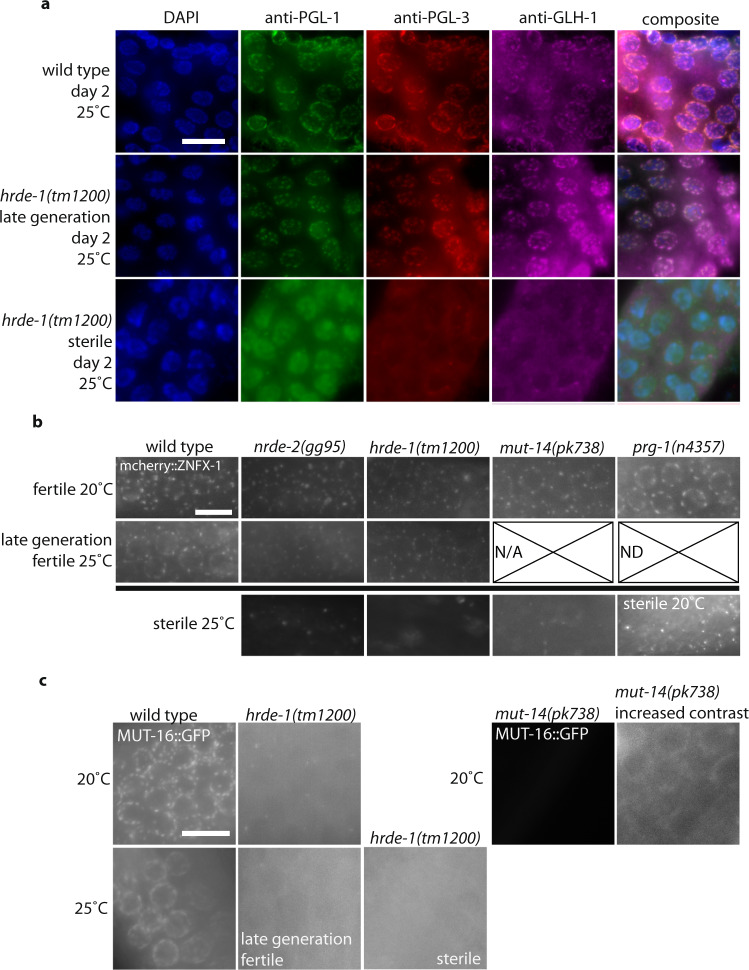
Loss of germ granules in sterile Piwi mutants. **a** Germlines of wild-type (*n* = 4), late-generation fertile (*n* = 4), and sterile *hrde-1* animals (*n* = 4) were stained as day 2 adults using antibodies against the P granule proteins PGL-1 (green), PGL-3 (red), and GLH-1 (magenta). Two independent PGL-1, PGL-3, and GLH-1 immunofluorescence experiments were performed with similar results using wild-type and mutant animals. Representative images are shown. Scale bar indicates 10 µm. **b** *nrde-2, hrde-1, mut-14*, and *prg-1* mutants containing mCHERRY::ZNFX-1 were grown at 20 °C or 25 °C as indicated. Germlines of day 2 (25 °C) and day 3 (20 °C) are shown for late-generation fertile and sterile animals. Sterile animals show mCHERRY::ZNFX-1 abnormalities. Two independent imaging experiments were performed and representative images are shown. Scale bar indicates 10 µm (20 °C: wild type *n* = 3; *nrde-2* *n* = 4, *hrde-1* *n* = 11; *mut-14* *n* = 5; *prg-1* fertile *n* = 6; *prg-1* sterile *n* = 5. 25 °C: wild type = 4; *nrde-2* fertile = 5; *nrde-2* sterile = 2; *hrde-1* fertile = 11, *hrde-1* sterile = 4; *mut-14* sterile = 8). **c** *hrde-1* and *mut-14* mutants containing MUT-16::GFP were grown at 20 °C or 25 °C as indicated. Germlines of day 2 (25 °C) and day 3 (20 °C) are shown for late-generation fertile and sterile animals; *hrde-1* and *mut-14* already show MUT-16::GFP reductions compared to wild type, while the animals are fertile at 20 °C. An image with increased contrast was included for *mut-14* to indicate that trace amounts of MUT-16::GFP are still present. Two independent imaging experiments were performed and representative images are shown. Scale bar indicates 10 µm (20 °C: wild type *n* = 4; *hrde-1* *n* = 4; *mut-14* *n* = 5; 25 °C: wild type *n* = 7; *hrde-1* fertile *n* = 3 *hrde-1* sterile *n* = 4).

We asked if P granule proteins other than PGL-1 were perturbed in late-generation fertile and sterile *hrde-1* mutants. We observed loss of the P granule components PGL-3 and GLH-1 in sterile *hrde-1* mutants compared to late-generation fertile animals (Fig. 3a), suggesting that P granule integrity may be strongly compromised in sterile Piwi pathway mutants. Z granules are components of *C. elegans* germ granules that intermix with and occur adjacent to P granules and Mutator foci^30,31^. We studied an epitope-tagged Z granule component mCHERRY::ZNFX-1 in *hrde-1*, *nrde-2*, *mut-14*, and *prg-1* mutants and observed decreased mCHERRY::ZNFX-1 localization, while fertile late-generation siblings had mCHERRY::ZNFX-1 localization similar to wild type (Fig. 3b). We studied Mutator foci and found that MUT-16::GFP levels were already reduced at 20 °C in fertile *hrde-1* and *mut-14* mutant adult germlines in comparison to wild type, while sterile *hrde-1* mutants lacked visible MUT-16 mutator foci (Fig. 3c). This suggests that Mutator foci are highly sensitive to perturbation of the Piwi/piRNA and secondary siRNA biogenesis pathways.

Several examples of *C. elegans* mutants with genome silencing defects that become immediately sterile are known. *Mutator* mutants experience strong levels of transposon desilencing and remain fertile indefinitely at low temperatures^8^ but display immediate fertility defects at 25 °C^41^. We examined germlines of sterile *mut-14* mutant adults at 25 °C and observed pronounced P granule defects in 85% of animals that lacked offspring or laid eggs that could not develop into adults (Fig. 2b and Table S1). We also examined mutants deficient for the HP1 protein HPL-2, which promotes heterochromatin maintenance and responds to Piwi/piRNAs; *hpl-2* mutants are frequently infertile at 25 °C^42^. PGL-1 localization was partially lost in fertile (33%, *n* = 11) and to a larger extent in sterile 2-day-old *hpl-2* adults (93%, *n* = 10) (Fig. S2 and Table S2). Similarly to Piwi pathway mutants grown at 20 °C, some 25 °C *hpl-2* mutant L4 larvae (47%, *n* = 17) exhibited germlines lacking PGL-1 expression (Fig. S2 and Table 2).

We asked if germ granules are affected in mutants that become sterile as a consequence of telomere dysfunction. We studied *trt-1* mutants that are defective for telomerase but maintain their telomeres via the telomerase-independent alternative lengthening of telomeres (ALT) pathway if grown under crowded conditions but become sterile if grown under low population densities^43,44^. We found that neither fertile nor sterile *trt-1* mutant ALT adults displayed germ cells with aberrant localization of the P granule components PGL-1, PGL-3, or GLH-1 (Fig. S3).

### Depletion of germ granule proteins phenocopies Piwi mutant sterility

**Fig. 4:**
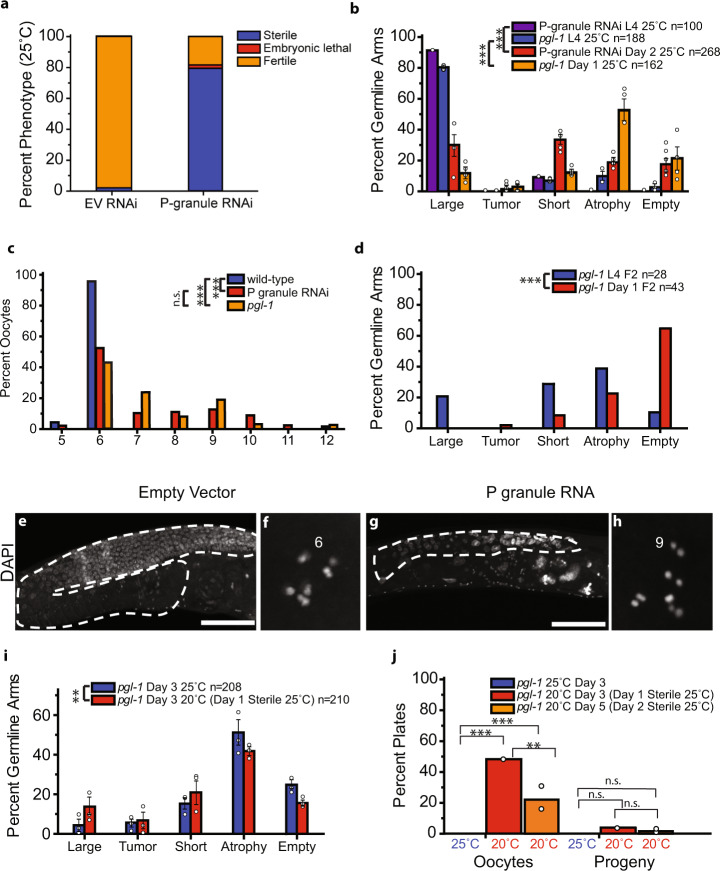
Disruption of P granules by RNAi or by deficiency for *pgl-1* results in similar phenotypes. **a** F2 wild-type (N2) worms on Empty Vector (EV) or P granule RNAi at 25 °C were scored for sterility, embryonic lethality, or fertility; *n* = 16–30 worms per condition, two independent experiments. **b** Germline phenotypes of 25 °C exposed F1 L4 *pgl-1* mutants and F2 L4 P granule RNAi-treated animals stained by DAPI. **c** F1 *pgl-1* mutants and F2 P granule RNAi-treated animals contained oocytes with increased numbers of univalents at sterility as measured by DAPI bodies, *n* = 151 wild type, 81 P granule RNAi, 37 *pgl-1*. Significance was determined between wild-type, P granule RNAi, and *pgl-1* datasets. **d** Stained DAPI germlines of sterile F2 L4 and day 1 *pgl-1* mutants. Significance was determined between *pgl-1* L4 and day 1 datasets. **e**–**h** Representative DAPI images of germline and oocyte defects in empty-vector-treated animals (**e**, **f**) and P granule RNAi-treated animals (**g**, **h**), scale bar = 100 µm. P granule RNAi-treated animals have a smaller germline than on control empty vector RNAi. At least four independent imaging experiments were performed and representative images are shown. While empty vector RNAi wild-type oocytes contain six univalents, oocytes following P granule RNAi treatment often have more than six univalents. **i** *pgl-1* F1 worms at 25 °C were either DAPI stained as day 3 adults (blue) or shifted to 20 °C on day 1 and DAPI stained at day 3 (red). **j** No sterile day 1 *pgl-1* F1 at 25 °C laid oocytes or progeny (*n* = 35 plates, 350 worms). Sterile F1 day 1 *pgl-1* shifted to 20 °C (*n* = 50 plates, 500 worms) and day 2 *pgl-1* shifted to 20 °C (*n* = 121 plates, 121 worms) gave rise to plates with oocytes and rarely progeny. Plates with oocytes contained both oocytes and dead embryos. Plates with progeny contained embryos that hatched to L1. For **b**, **d**, **i**, ***p < 0.0001, **p = 0.007 two-tailed Fisher’s Chi Square test with Bonferroni correction, *n* = total germline arms scored, for **c** and **j** ***p < 0.0001, *p < 0.01, n. s. = *p* > 0.05 two-tailed Fisher’s exact test for count data with Bonferroni correction, error bars are S.E.M.

As all three compartments of *C. elegans* germ granules were severely perturbed in most sterile Piwi mutants, we asked if germ granule dysfunction might be sufficient to induce phenotypes characteristic of Piwi mutant sterility. We initially used a previously developed RNAi strategy that simultaneously targets four P granule subunits *pgl-1*, *pgl-3*, *glh-1*, and *glh-4* (Fig. 4a)^45,46^. This resulted in many second generation (F2) sterile adults at 25 °C (80%) (Fig. 4a). The germlines of F2 P granule RNAi L4 larvae were mostly normal in size (~90%) in comparison to control worms. However, pronounced germ cell atrophy often occurred as P granule-depleted animals matured into sterile adults, which resulted in a significant change in their germline profile with significantly more shorter, atrophied, and empty germlines (Fig. 4b, *p* = 3.93E–24). This indicates that germline atrophy occurs as P granule-deficient L4 larvae mature into young adults. We also noted that a very small number of day 2 P granule RNAi had tumorous germlines (Fig. 4b). These small germline tumors contained some mature gametes but had mitotic cells in both distal and proximal segments of the germline^47^. Univalent chromosomes were previously observed in sterile but not fertile late-generation *rsd-6* mutant oocytes^11^. We found that 45.5% of oocytes of sterile P granule-depleted adults contained 7–12 univalents (Fig. 4c, e–h).

To confirm the sterility induced by P granule RNAi, we studied a *pgl-1* loss-of-function mutant that is fertile at low temperatures but displays maternal effect sterility if shifted to the restrictive temperature of 25 °C^48^. Most first-generation F1 *pgl-1* mutant animals at 25 °C displayed normal germlines at the L4 larval stage, but there was a significant shift in their germline profiles as 52% of day 1 adults showed germline atrophy (Fig. 4b, *p* = 1.04E−35, Table S3). Furthermore, when oocytes of sterile *pgl-1* mutant adults were scored, 56.7% of oocytes had univalents (Fig. 4c). Germline atrophy and univalent chromosome phenotypes are consistent with those of sterile late-generation Piwi pathway mutant adults^21^.

Fertile F1 progeny of *pgl-1* mutants whose parents were shifted from 20 °C to 25 °C gave rise to F2 animals that were uniformly sterile at 25 °C (*n* = 350). L4 stage F2 *pgl-1* mutants exhibited germline profiles with more severe germ cell proliferation defects than those observed for F1 L4 larvae, and F2 day 1 adults showed a striking 54% increase in empty germlines (Fig. 4d, *p* = 2.44E−05). Therefore, germlines of 25 °C F2 generation *pgl-1* mutant larvae displayed more pronounced germ cell proliferation defects than F1 progeny of *pgl-1* mutant mothers that were shifted to the restrictive temperature of 25 °C.

Sterile *prg-1*/Piwi mutant adults can regrow their atrophied germlines and become fertile^21^. To ask if germ granule dysfunction might elicit this reproductive arrest phenotype, we shifted sterile *pgl-1* mutant adults from the restrictive temperature of 25 °C to the permissive temperature of 20 °C. We isolated L4 larvae and waited a day until they were 1-day-old adults, because wild-type young adults of that age, cultured under the same conditions, were nearly always fertile (99.2%, *n* = 120), as evidenced by the presence of embryos within the uterus. In contrast, few first-generation F1 *pgl-1* mutant 1-day-old adults cultured at 25 °C possessed in utero embryos (5.67%, *n* = 159). We selected apparently sterile 1-day-old *pgl-1* mutant adults that lacked embryos to fresh plates and continued to culture one cohort at 25 °C while a second cohort was shifted to 20 °C. After 48 h, both cohorts of now 3-day-old adults were scored for fertility. Both cohorts were then maintained at 20 °C for an additional 3 days and scored for fertility once again as 6-day-old adults. We found that apparently sterile *pgl-1* mutants maintained continuously at 25 °C until they were 3-day-old adults invariably failed to become fertile. Moreover, downshift of these 3-day-old adults to 20 °C failed to restore fertility, resulting in completely sterile 6-day-old adults (Figs. 4j and S5a). In contrast to the apparently sterile 1-day-old adults that were kept at 25 °C, 48% of the apparently sterile *pgl-1* mutant adults that were downshifted to 20 °C as 1-day-old adults had laid oocytes and some additionally had embryos (4%) after 2 days at 20 °C (Fig. 4j). A further 15% (30% of the remaining 50% sterile) of these animals gained fertility during day 3 to day 6 of adulthood (Fig. S5a), with some embryos hatching into viable larvae. Consistently, apparently sterile *pgl-1* mutant 1-day-old adults that were downshifted to 20 °C exhibited significantly enlarged germlines as 3-day-old adults (*p* = 0.007) (Fig. 4i). We also scored progeny of sterile 25 °C F1 *pgl-1* adults that were downshifted to 20 °C as 2-day-old adults; 23% of these animals (*n* = 121) became fertile by laying oocytes or embryos, of which some developed into viable larvae (Fig. 4j).

These findings indicate that during early young adulthood *pgl-1* mutants, like *prg-1* mutants^21^, can recover fertility upon a return to permissive environmental conditions. For clarity, we summarize the results of this paragraph in a schematic figure (Fig. S6).

### Transcriptional consequences of germ granule dysfunction and Piwi pathway mutant sterility

The immediate sterility of Piwi/piRNA mutants in other species correlates with expression of transposons and associated genome instability^14,15,17,49^. We therefore studied RNA from the nuclear RNAi defective mutants *nrde-1*, *nrde-2*, and *nrde-4*^32,50,51^. Although the *nrde-2* mutant only becomes sterile at 25 °C, *nrde-1* and *nrde-4* mutants become progressively sterile at both 20 °C and 25 °C^33^. In addition, *nrde-1* and *nrde-4* mutants have relatively large brood sizes just prior to onset of late generation sterility. We did not use *prg-1* mutants in these experiments because they develop very low brood sizes in late generations^21^ making it difficult to isolate sufficient numbers of synchronous sterile *prg-1* mutant animals. We prepared RNA from synchronized early-generation (fertile) and late-generation (sterile) *nrde-1* and *nrde-4* mutants maintained at 20 °C and compared their mRNA profiles to those of *nrde-2* mutant and wild-type controls that remain fertile indefinitely at 20 °C.

**Fig. 5:**
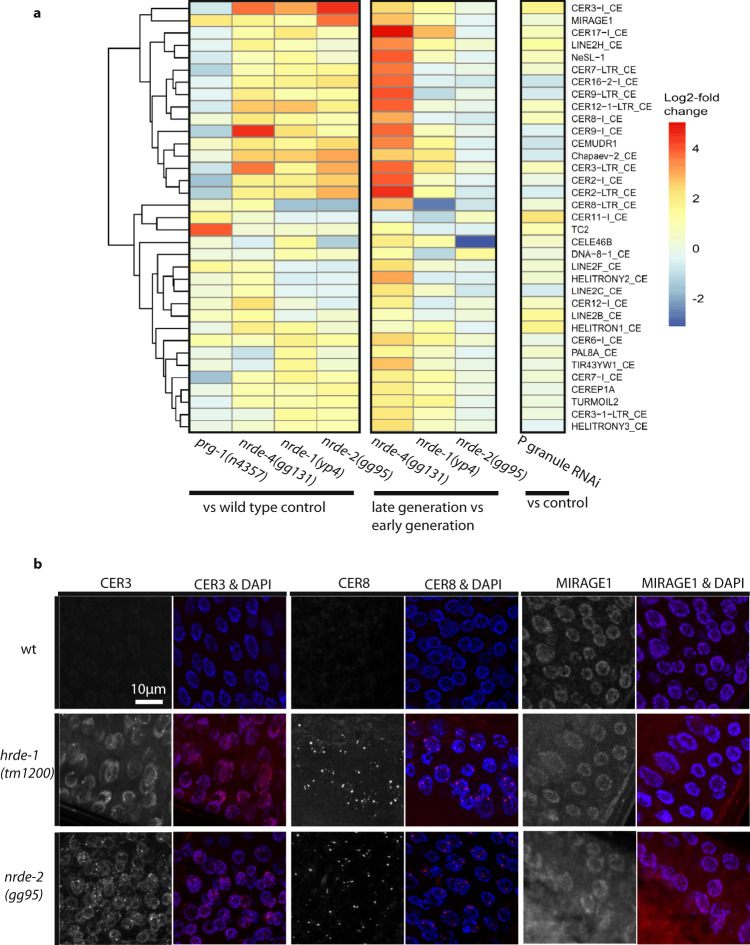
Transcriptome profiling of germ granule deficient larvae of Piwi pathway genome silencing mutants *nrde-1* and *nrde-4*. **a** Log2-fold changes in transposon transcript abundance in mutant vs wild type, late vs early generation, or P granule RNAi-treated vs control. Transposons upregulated at least twofold in *nrde-1*, *nrde-4*, or *prg-1(n4357)* are shown. **b** Single molecule RNA FISH signals for CER3, CER8, and MIRAGE1 elements in the adult germline. More than seven animals were examined in each group from two experimental datasets showing similar results and representative images are displayed (CER3: wild type *n* = 8; *hrde-1* *n* = 7; *nrde-2* *n* = 20 CER8: wild type *n* = 15; *hrde-1* *n* = 13; *nrde-2* *n* = 13; MIRAGE1: wild type *n* = 10; *hrde-1* *n* = 13; *nrde-2* *n* = 12).

Sterile generation *nrde-1* and *nrde-4* mutant L4 larvae showed strong upregulation of a common set of transposon classes that were not upregulated in early-generation fertile animals or in wild-type controls (Fig. 5a). We identified 18 transposon classes that were upregulated at least twofold in both *nrde-1* and *nrde-4* (CEMURD1, CER-12-LTR, CER16-2-I, CER17-I, CER2-I, CER2-LTR, CER3-I, CER-3-LTR, CER7-I, CER7-LTR, CER8-I, CER9-I, CER9-LTR, CEREP1A, Chapaev-2, HELITRON1, MIRAGEI, TURMOIL1) 13 of which were CER retrotransposons, consistent with previous transgenerational analysis of *hrde-1* mutants^52^. However, we also found that *nrde-2* mutant controls that do not become sterile at 20 °C displayed strong upregulation of similar transposon loci, even in early generations. Of the 18 transposons upregulated in both *nrde-1* and *nrde-4*, 17 were also upregulated in *nrde-2* compared with wild type (all but HELITRON1; Fig. 5a). Previous work reported that distinct classes of transposons are upregulated in *prg-1* and *nrde-2* mutants^8,18,36,52^. Consistently, we analyzed published RNA-seq data from *prg-1(n4357)* mutants^18^ and found that MIRAGE1 was the only transposon commonly upregulated in *nrde-1*, *nrde-4*, and *prg-1* mutants^18^ (Fig. 5a). Therefore, although *prg-1*, *nrde-1*, and *nrde-4* mutants are all defective for small RNA-mediated genome silencing and germ cell immortality, these genes have largely distinct transposon silencing functions.

To ask if transposon expression could be detected in germline or somatic cells, we used RNA FISH to examine late-generation *hrde-1* and *nrde-2* mutants, which only become sterile if grown at 25 °C, for the presence of the CER3, CER8, and MIRAGE1 transposable element RNA. *hrde-1* and *nrde-2* mutants had elevated CER3 and CER8 expression compared to wild type, while MIRAGE1 was visible in the tested mutants as well as wild-type germlines at a similar level (Fig. 5b). This indicates that that MIRAGE1 is upregulated in wild type at the elevated temperature of 25 °C. In addition to high germline expression, MIRAGE1 was also highly upregulated in the soma of late-generation *nrde-2* mutants grown at 25 °C, but we failed to observe somatic expression of other transposons in any of the mutant backgrounds tested (Fig. 5b). Overall, these RNA FISH studies suggest that transposon upregulation is mostly limited to germ cells.

In addition, we found that late-generation *prg-1* mutant^18^ and sterile generation *nrde-1* and *nrde-4* mutant gene expression profiles were very different from those observed for wild-type or *nrde-2* controls (Fig. S4a and b). Spermatogenic genes were over-represented among genes significantly upregulated in sterile generation *nrde-1* and *nrde-4* mutant larvae (*p* = 1e−15, Chi Square test for both mutants), which is consistent with what has been previously reported for different chromatin mutants like *spr-5* and *rsd-2* that become progressively sterile^11,53,54^. Recently, Rogers and Phillips^55^ also found spermatogenesis genes significantly upregulated in *mut-16* mutants whose fertility is compromised at restrictive temperature but also in wild-type controls. We suggest that spermatogenesis gene upregulation might be a common property that is associated with Piwi pathway mutant sterility in *C. elegans*.

In order to identify signature changes in gene expression relevant to late-generation sterility of Piwi pathway mutants, we searched for genes that are co-regulated in several mutants. We identified only 11 genes that were significantly upregulated and no common genes that were significantly downregulated for *prg-1*, *nrde-1*, and *nrde-4* mutants. This suggests that *prg-1* and *nrde-1/nrde-4* mutants have fundamentally different gene expression profiles at or near sterility (Fig. S4a). Furthermore, 20 genes were significantly upregulated at least fourfold in *prg-1* and either *nrde-1* or *nrde-4*, including four *pud* (protein upregulated in dauer) genes (Table S4). Dauer formation occurs in response to various stresses, and adult reproductive arrest can also be induced in response to stress^56,57^. Therefore, *pud* gene expression could reflect a general stress response that is associated with distinct forms of developmental arrest.

Previously, a strong correlation for histone gene downregulation in *prg-1* mutants was observed^58^ and we found strong histone gene downregulation for a distinct *prg-1* mutant dataset^18^ (Fig. S4c). However, we did not see consistent changes for histone gene expression in our late-generation *nrde-1* and *nrde-4* mutants (data not shown). In addition, we could not find consistent changes in any gene whose knockdown perturbs P granule structure^27^, suggesting post-transcriptional regulation of germ granule structure for sterile Piwi pathway mutants.

RNA-seq datasets were previously obtained for germlines of P granule defective L4 larvae (animals destined to become sterile adults), but gene expression was not significantly different from wild type^45^. We compared RNA from germlines of P granule defective L4 larvae^45^ with RNA from sterile generation *nrde-1* and *nrde-4* mutant L4 larvae and found no genes that were up- or downregulated in both *nrde-1/nrde-4* and P granule-defective larvae. Moreover, only two of 18 transposons upregulated in *nrde-1/nrde-4* were also upregulated in P granule defective L4 larvae (Fig. 5a). We conclude that although simultaneous knockdown of four P granule components leads to germline degeneration phenotypes that mimic those of sterile generation Piwi pathway mutants, this does not cause immediate overt effects on expression of heterochromatic segments of the genome or on genes in a manner that mimics the transcriptomes of Piwi pathway mutants that are poised to become sterile.

These findings indicate that although the phenotypes of worms subjected to P granule RNAi exhibit many similarities to those of late- or sterile-generation *prg-1* and *nrde-1,4* mutant worms those similarities do not extend to their transcriptome profiles. Moreover, we could not identify a common transposon or group of transposons that could explain their sterility. While this manuscript was in review, several other groups concluded that transposon upregulation is unlikely to be a primary cause of sterility in mutants deficient for small RNA-mediated genome silencing^55,58,59^.

### Deficiency for *pgl-1* enhances *prg-1*/Piwi mutant sterility

**Fig. 6:**
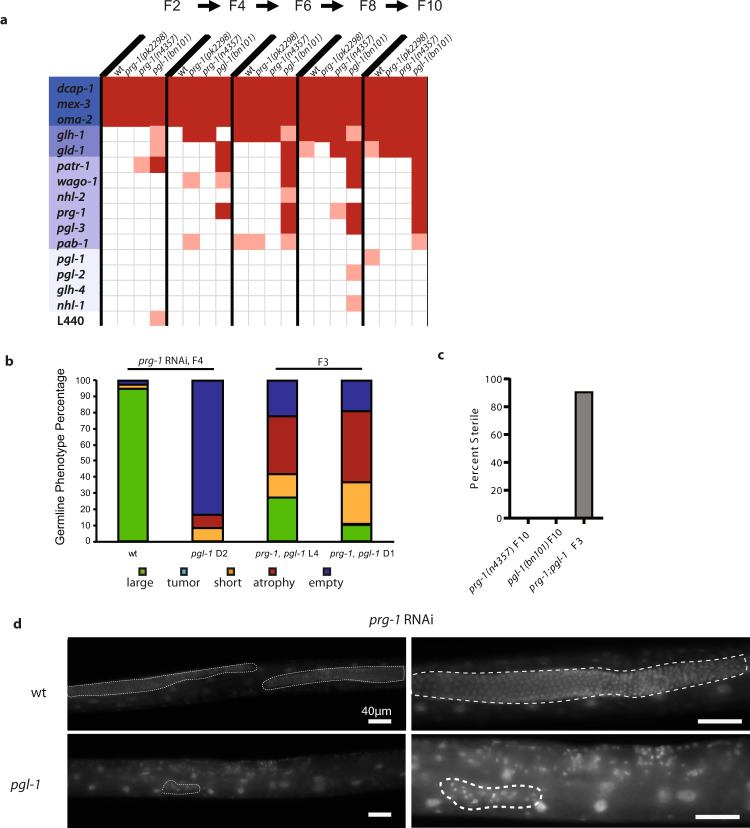
Deficiency for *pgl-1* results in rapid sterility of *prg-1* mutants. **a** Germline immortality assay with wild type, *prg-1(pk2298), prg-1(n4357)*, and *pgl-1(bn101)* on different P granule RNAi clones. Two replicates of three L1 larvae were transferred every week or after two generations and plates were scored as sterile when no more than three worms were present after 1 week. Light red indicates sterility for 1 of 2 replicate plates while dark red indicates sterility for both replicates. When one plate became sterile, worms from the second plate were used for two replicates. Different shades of blue were used to visually group different RNAi datasets that showed a similar sterility pattern for wt, *prg-1*, and *pgl-1* worms. **b** Germline sizes of wild type and *pgl-1(bn101)* on *prg-1* RNAi after four generations (wt *n* = 39; *pgl-1* *n* = 12) and of *prg-1; pgl-1* double mutant F3 animals (*n* = 40); **c** *prg-1; pgl-1* double mutants display marked sterility in the F3 generation. **d** Germline comparison after DAPI staining of wild type and *pgl-1(bn101)* on *prg-1* RNAi after four generations. The germline arms are outlined with a white dotted line. While two germline arms could be detected in wild type that are large and visible throughout the body, only a small group of germ cells was detected in *pgl-1(bn101)*. At least 12 different animal germlines were examined in wild type and F4 *pgl-1(bn101)* on *prg-1* RNAi, and representative images are shown.

We next tested for synthetic interactions between *prg-1* and known P granule factors. To do this, we used RNAi to knock down several genes whose protein products localize to P granules in early-generation *prg-1* mutants or in *pgl-1* mutants grown at 20 °C, which is the permissive temperature for *pgl-1* mutants. We included *prg-1(RNAi)* in this assay as a control. As expected, several genes known to be essential for fertility caused immediate first-generation sterility, verifying the effectiveness of our RNAi conditions (Fig. 6a). RNAi of *prg-1* induced sterility in the *pgl-1* mutant in generation F4 (Fig. 6a, b). Sterile *pgl-1* mutants treated with *prg-1* RNAi had a mixture of empty, atrophied, and short germlines (Fig. 6b, d). Knockdown of the germ granule components *gld-1* and *pgl-3* also induced F4 generation sterility for *pgl-1* mutants (Fig. 6a), perhaps consistent with a common function for PRG-1, GLD-1, and PGL-3 proteins that are known to physically interact^60,61^.

To confirm the above results, we used outcrossed *prg-1* and *pgl-1* mutant worms and created *prg-1; pgl-1* double mutants. We found that as single mutants *prg-1* (*n* = 20) and *pgl-1* (*n* = 20) were fertile for ten generations when grown at 20 °C. In contrast, *prg-1; pgl-1* double mutant F2 animals were fertile but gave rise to F3 adults that were almost all sterile (41/45) (Fig. 6c). We stained sterile *prg-1; pgl-1* double mutant F3 day 1 adults with DAPI and found that 62.9% of germlines were either atrophied or empty (Fig. 6b).

When *pgl-1* mutants were grown for 14 generations at 20 °C, we found that about 40% of the lines became sterile. This *pgl-1* germline mortality phenotype resembles that previously described for *prg-1* (12% sterility in F14; Fig. S5b, ref. ^8^). The *prg-1* mutant Mortal Germline phenotype can be rescued by *daf-2* mutation (Fig. S5b, ref. ^8^). However, we found that *pgl-1* germline mortality was not rescued by a *daf-2* mutation (Fig. S5b). The rapid transgenerational sterility of *pgl-1* mutants that are deficient for *prg-1* indicates that PRG-1 and PGL-1 play distinct roles in germ cell immortality. Functional interplay between these proteins could be reflected by their potential physical interaction^58^.

## Discussion

Previous work has demonstrated that the immediate sterility of Piwi mutants in several species correlates with transposon expression, transposition, and genomic instability^14,15,17^. Although this suggests that DNA damage might cause Piwi mutant sterility, we previously found that *C. elegans* mutator mutants with high levels of transposition in their germlines can be grown indefinitely^8^. A recent study revealed that genome silencing defects caused by *met-2* and *set-25* H3K9 histone methyltransferases causes R-loops and mutations at tandem repeats in the *C. elegans* genome, reduced fertility, and synthetic sterility with knockdown of the ATR homolog *atl-1* (ref. ^20^). Although MET-2 and SET-25 may promote genome silencing in response to Piwi/piRNAs, we show here that the sterility of the Piwi mutants is not consistently associated with high levels of ATM/ATR DNA damage signaling. Consistently, we found that telomerase deficiency causes a distinct form of transgenerational sterility that is likely to be the consequence of telomere-related DNA damage and occurs independently of germ granule defects that are common in sterile Piwi mutants (Figs. S3 and 6c).

We discovered defects in three distinct germ granule compartments that were markedly pronounced in sterile late-generation Piwi pathway mutants in comparison to fertile Piwi mutant siblings that had diminished levels of fertility and would become sterile within a few generations (Figs. 2, 3, S1, S2, and 7a, b). Our findings suggest that the collapse of germ granule domains begins with Mutator foci that become faint when Piwi pathway mutants are still fertile (Fig. 3c), whereas prominent effects on P and Z granule structure are restricted to the sterile generation (Fig. 3a, b and Table S1). We observed strong germ granule dysfunction for sterile Piwi mutants at the L4 stage of development, when the initial effects on germ cell proliferation are observed in sterile Piwi mutants^21^. If germ granule dysfunction is the earliest defect that occurs in the context of Piwi mutant sterility, analysis of germ granule structure prior to the L4 larval stage might reveal the developmental stage at which Piwi mutants decide to become sterile.

**Fig. 7:**
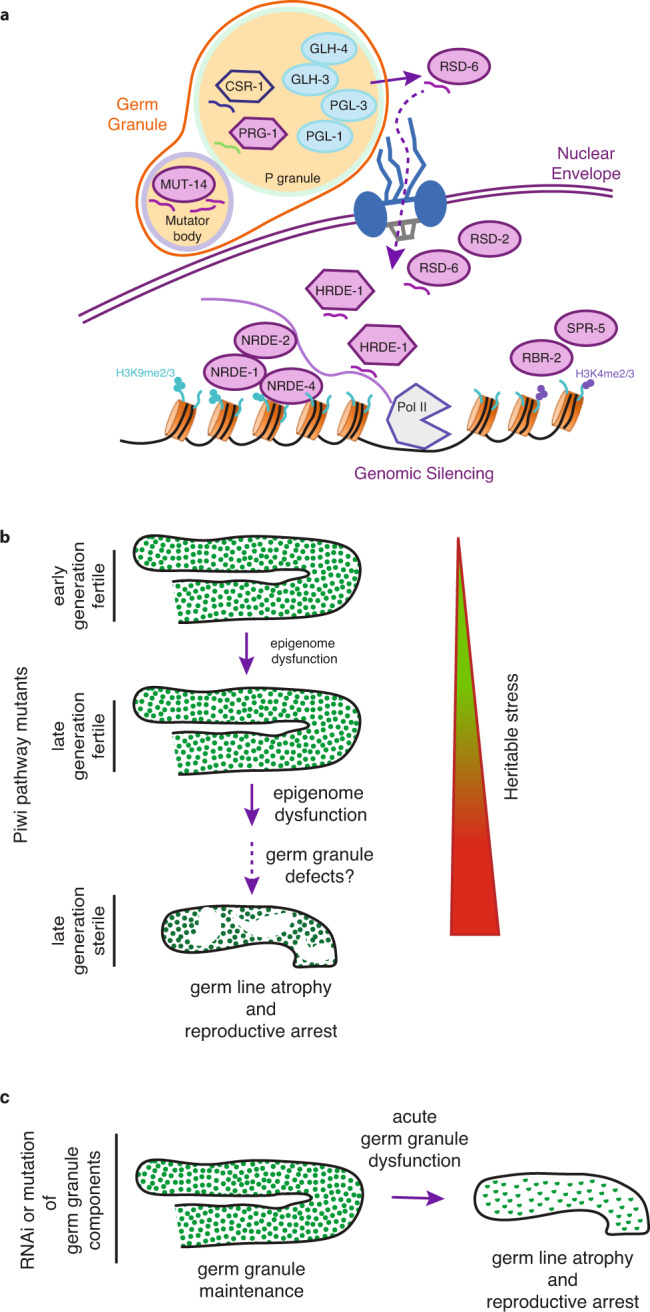
Model for P granule dysfunction in Piwi pathway genome silencing mutants. **a** Model of P granules and associated small RNA-mediated genome silencing proteins. Blue circles contain P granule components analyzed in this paper. Mutants used in this paper are in lavender. Argonaute proteins are hexagons. Simplified representation of the small RNA pathways relevant to this manuscript: under wild-type conditions, small anti-sense RNAs are produced for all mRNAs inside germ granules that consist of the core proteins PGL-1, PGL-3, GLH-1, and GLH-4. Those anti-sense RNAs are bound by the Argonaute proteins CSR-1 or PRG-1 (or WAGO-1, not shown) and allow mRNA sorting. CSR-1 bound mRNAs are licensed for translation while PRG-1 bound mRNAs will be silenced. Thus, PRG-1 binding allows for the transcription of secondary siRNAs inside mutator bodies. MUT-14 is a DEAD box protein that is essential for the production of secondary siRNAs. RSD-6 and HRDE-1 may help to transport the secondary siRNAs back into the nucleus where they bind to nascent RNA transcripts. Binding of the siRNA transcripts to the nascent RNA transcripts will lead to gene silencing through the recruitment of the NRDE-1/2/4 complex and subsequently the recruitment of histone modifying enzymes like the histone demethylases RBR-2 and SPR-5. **b** Model for transgenerational sterility in response to accumulation of heritable stress in Piwi pathway genome silencing mutants, which ultimately compromises P granule integrity: In early generations, germline are fertile and contain germ granules evenly surrounding germline nuclei. In late-generation small RNA mutants, the epigenetic stress caused by failing RNA silencing pathways (heritable stress) will then result in dysfunctional germ granules and reproductive arrest in PIWI pathway mutants. Dotted arrow indicates a correlative relationship. **c** Model for induction of reproductive arrest by acute dysfunction of P granule components: Similarly, to **b** RNAi or the mutation of P granule core components causes the same phenotype as observed for PIWI pathway mutants.

To assess the functional significance of pronounced germ granule defects in sterile Piwi pathway mutants, we induced germ granule dysfunction by RNAi knockdown of four P granule proteins and by mutation of the P granule component *pgl-1*. We observed a broad range of germline phenotypes that occur in sterile late-generation *prg-1*/Piwi pathway mutants (Figs. 4 and 7c)^21^. It has been previously proposed that misrouting of small RNAs associated with pro- and anti-silencing small RNA pathways could be the cause of sterility in Piwi pathway genome silencing mutants^62,63^. This agrees with our observations of the collapse of germ granules in sterile late-generation Piwi mutants and also agrees with a recent study by Rogers and Phillips that found that perinuclear PGL-1 is lost when heat stress induces sterility of *mut-16* mutants that are deficient for secondary siRNA biogenesis^55^. Germ granule defects may be a common theme of metazoan Piwi sterility^14,15,17^, because they also occur in mouse mutants that become sterile as a consequence of piRNA biogenesis defects^64,65^.

There are several possible explanations for our findings. First, the germ granule defects of sterile Piwi mutants could be a consequence rather than a cause of Piwi sterility. Second, although P granule dysfunction was able to phenocopy Piwi sterility phenotypes, P granule dysfunction could induce a stress that activates the reproductive arrest program of Piwi mutants, independent of germ granule collapse. Third, sterile *pgl-1* mutants could be in a state of developmental arrest that is distinct from the reproductive arrest program that occurs when Piwi mutants become sterile. Fourth, germ granule dysfunction might cause Piwi mutant sterility. Whatever the explanation for the inherently variable form of reproductive arrest that occurs in sterile Piwi mutants, including germline atrophy, empty germlines, germline tumors, univalent chromosomes in oocytes, germline regrowth, and restoration of fertility, we are not aware of another *C. elegans* mutant that causes sterility and displays this broad spectrum of phenotypes. The fact that many, if not all, of these phenotypes occur in response to acute germ granule dysfunction is consistent with the hypothesis that Piwi mutant sterility might be a reproductive arrest program that is driven by germ granule dysfunction. This hypothesis, which remains to be proven, is appealing even if it might be difficult to address experimentally. Although not currently feasible, one could attempt to rescue Piwi mutant sterility by re-introducing germ granules isolated from wild-type animals into sterile Piwi mutant germlines.

We uncovered little overlap in transcriptional profiles of sterile animals that were deficient for Piwi or germ granules, even though these animals displayed similar germline phenotypes (Fig. 6). This might mean that reproductive arrest in response to these defects is triggered by distinct transcriptional programs with distinct causes: Piwi mutant sterility and germ granule dysfunction. Alternatively, lack of a common transcriptional response for Piwi and germ granule defective mutants could suggest that a common post-transcriptional mechanism promotes reproductive arrest when Piwi or germ granule defects induce sterility. This post-transcriptional hypothesis might be consistent with the notion that germ granules regulate the fate of mRNAs exported from the nucleus to the cytoplasm, so germ granule dysfunction could lead to accumulation of aberrant cytoplasmic transcripts. Furthermore, stress granules and P bodies are cytoplasmic compartments that share components with germ granules and promote rapid post-transcriptional RNA-mediated responses to cellular stress^66^. Consistent with the possibility that Piwi pathway mutant sterility might be a post-transcriptional reproductive arrest mechanism (Fig. 4i, j and S5a), we failed to detect misregulation of genes known to be required for P granule formation (Fig. S4)^27^.

It has previously been shown that PGL-1::GFP disappears at P granules upon inhibition of transcription^67^, while distinct germ granule components remain. Hence, changes to the distribution of RNA polymerase within a nucleus might trigger loss of some germ granule structures. If Piwi/piRNA silencing defects significantly alter the transcriptional landscape within germ cell nuclei, this might create a signal that is created by the cellular response to inhibition of transcription.

Consistent with our study, recent analyses of transcription in small RNA-mediated genome silencing mutants revealed that transposon expression may not cause the progressive sterility of *C. elegans* small RNA mutants^55,58,59^. Reed et al. found downregulation of histone genes in *prg-1* mutants^59^, similarly to Barucci et al.^58^, but they did not observe increased downregulation in later generations. We observed similar histone mRNA downregulation in independent *prg-1* mutant transcriptional datasets^18^ (Fig. S4c) but did not find similar histone mRNA changes in *nrde-1* and *nrde-4* mutants that are poised to become sterile. The potential relationship of histone gene expression and Piwi mutant sterility remains an intriguing question.

Spermatogenesis and oogenesis genes were the most upregulated category of genes in *prg-1* and *mut-16* mutants in previously published work^55,58,59^, and this was also true for our *nrde* mutant datasets. Increased expression of spermatogenesis genes was also observed for *rsd-2* mutants^11^ and for *spr-5* and *met-2* mutants^53,54^. Although Rogers and Phillips also found specific upregulation of spermatogenesis genes in wild type at 25 °C compared to 20 °C, this was only slightly higher in *mut-16* mutants at the same temperature^55^. Therefore, Rogers and Phillips concluded that spermatogenesis genes might shift their chromatin state more easily than other regions in the genome^55^, which could be relevant to induction of sterility in Piwi mutants.

It is possible that germ granules are required for, or form in concert with or as the consequence of small RNA surveillance pathways that function in these perinuclear granules^68,69^. Although simultaneous depletion of four P granule factors is sufficient to induce phenotypes that occur in sterile late-generation Piwi mutants, this does not lead to de-silencing of transposons. If existing genomic silencing marks are at least partially maintained in the absence of small RNAs, as previously suggested^9^, this might mitigate the effects of germ granule collapse on heterochromatin that is created in response to small RNAs.

Components of germ granules that suppress transposons and viruses are likely to be frequently challenged by these parasitic elements^27^. Our results establish a novel framework for considering Piwi mutant infertility. We speculate that metazoan germlines have evolved a reproductive arrest mechanism that enables fertility to be preserved in response to Piwi pathway dysfunction. An initial component of this reproductive arrest mechanism might be activation of a sensor of Piwi dysfunction, which may not be DNA damage but might be another form of transcriptional or genomic stress that remains to be discovered. Once Piwi pathway dysfunction is sensed in late-generation *C. elegans* Piwi mutants, a reproductive arrest mechanism is triggered.

One of several explanations for the observations of our study is that the collapse of germ granules might orchestrate the reproductive arrest of sterile Piwi mutants. If this is the case, then this reproductive arrest mechanism could reflect a long-standing arms race between genome parasites and their hosts. It is possible that an unchecked attack by a parasitic element might be effectively countered by dismantling the machinery that is targeted by the parasitic element, much of which lies within germ granules. A reversible arrest in oogenesis could provide the germline time to adapt to formidable transposon or viral parasites by establishing a parallel genome silencing pathway that preserves genome integrity prior to restoration of fertility. In this context, analysis of small RNAs in diverse nematode species has revealed that RNA-dependent RNA polymerase and Dicer pathways have evolved to substitute for Piwi/piRNA genome silencing during nematode evolution^70^.

## Methods

### Strains

All strains were cultured at 20 °C or 25 °C on Nematode Growth Medium (NGM) plates seeded with *Escherichia coli* OP50. Strains used include Bristol N2 wild type, *hrde-1(tm1200) III, nrde-1(yp4) III, nrde-2(gg95) II, nrde-4(gg131) IV, prg-1(n4357) I, prg-1(tm872) I, prg-1(pk2298) I, pgl-1(bn101) IV, pgl-1(bn102) IV, pgl-1(bn101) IV prg-1(n4357) I, mut-14(pk738) V, rbr-2(tm1231) IV, hpl-2(tm1489) III, hpl-2(ok1061) III, his-72::mNeonGreen* (LP230) III, *mut-16::gfp I, hrde-1(tm1200) III mut-16::gfp I, mut-14(pk738) mut-16::gfp, mcherry::znfx-1 II, prg-1(n4357) I mcherry::znfx-1 II, nrde-2(gg95) II mcherry::znfx-1 II, hrde-1(tm1200) III mcherry::znfx-1 II, mut-14(pk738) V mcherry::znfx-1 II, prg-1(n4357) I daf-2(e1368) III, pgl-1(bn102) IV I daf-2(e1368) III, trt-1(ok410) I*.

### Mutants obtained by crossing

*pgl-1(bn102) daf-2(e1368), prg-1(n4357) daf-2(e1368), pgl-1(bn101) prg-1(n4357), hrde-1(tm1200) mut-16::gfp* and *mut-14(pk738) mut-16::gfp* were obtained by crossing. Double mutants were identified by PCR products, sensibility to heat stress, or fluorescence microscopy. Primers used are listed in Table S5.

### RNAi assay

Feeding RNAi plates harboring host bacteria HT115(DE3) engineered to express “quad” dsRNA (targeting *pgl-1*, *pgl-3*, *glh-1*, *glh-4*) were obtained from Susan Strome^45,46^. L1 larvae were placed onto freshly prepared feeding RNAi plates with dsRNA induced by 1 mM IPTG (isopropyl-β-d(-)-thiogalactopyranoside) and were transferred after one generation at 25 °C and collected at F2 adults as described^45^ for DAPI staining, oocyte and germline analysis.

For RNAi screen, L1 worms containing the *his-72*::*mNeonGreen* transgene were arrested in M9 media and then pipetted at a density of 20 worms per plate and maintained at 20 °C. At the L4 stage and during adulthood worms were scored for germline size using Leica M205 fluorescence microscope.

For the P granule germline immortality assay, three L1 worms were transferred every week on two replicate plates per condition at 20 °C. Plates were counted as sterile when no more than three worms were present after one week. Partial sterility was counted when one out of two plates became sterile while both replicate plates were sterile for full sterility.

### DAPI staining and scoring

DAPI staining was performed as previously described^3^. Briefly, L4 larvae were selected from sibling plates and sterile adults were singled as late L4s, observed 24 h later for confirmed sterility, and then stained 48 h after collection. Univalents were scored by counting DAPI bodies in the −1 to −4 oocytes. Germline profiles were scored using the method outlined in Heestand et al.^21^. In brief, we scored germline arms into five categories. Large germlines were comparable to wild-type controls. Tumorous germlines were similar to proximal proliferative tumors^71^. Short germlines were far shorter and underproliferated compared to both large germlines and wild-type controls but still contained meiotic cells. Atrophy germlines were a small population of germ cells (50–100) near the distal tip cell that were typically mitotic and devoid of meiotic germ cells. Empty germlines were devoid of germ cells.

### Statistical analysis

Statistical analysis was performed as previously described^21^. Briefly, statistical analysis was performed using the R statistical environment^72^. For germline phenotypes, contingency tables were constructed and pairwise Chi Square tests or Fisher’s exact test for count data with Bonferroni correction was used to determine significant differences in germline phenotype distributions. The significance of the DNA damage response analysis was determined by a Kruskal–Wallis test, followed by a Mann–Whitney test between individual samples; *p*-values were adjusted with Bonferroni correction when multiple comparisons were performed.

### RNA extraction and sequencing

Animals were grown at 20 °C on 60-mm NGM plates seeded with OP50 bacteria. RNA was extracted using Trizol (Ambion) followed by isopropanol precipitation. Library preparation and sequencing were performed at the UNC School of Medicine High-Throughput Sequencing Facility (HTSF). Libraries were prepared from ribosome-depleted RNA and sequenced on an Illumina Hiseq 2500.

### RNA-seq analysis

The following publicly available RNA-seq datasets were downloaded from the Gene Expression Omnibus (https://www.ncbi.nlm.nih.gov/geo/): GSE92690 (P granule RNAi experiment) and GSE87524 (*prg-1* experiment). Adapter trimming was performed as required using the bbduk.sh script from the BBmap suite (version 37.36)^73^ and custom scripts (see “Code availability” section). Reads were then mapped to the *C. elegans* genome (WS251) using hisat2 (version 2.1.0)^74^ with default settings and read counts were assigned to protein-coding genes using the featureCounts utility from the Subread package (version 1.5.3)^75^. For multimapping reads, each mapping locus was assigned a count of 1/*n* where *n* = number of hits. Differentially expressed genes were identified using DESeq2 (version 1.16.1), and were defined as changing at least twofold with FDR-corrected *p*-value <0.01. For analysis of transposon RNAs, reads were mapped to the *C. elegans* transposon consensus sequences downloaded from Repbase (http://www.girinst.org/repbase/) with bowtie (version 1.2.2)^76^ using the options -M 1 -v 2. Transposons with fewer than 10 counts in each sample were excluded from further analysis. Counts were normalized to the total number of mapped reads for each library for the *prg-1* dataset, or to the total number of non-ribosomal mapped reads for all other datasets. A pseudocount of 1 was added to each value to avoid division by zero errors. Analysis of sequencing data and plot creation was performed using the R statistical computing environment^72^. Clustering of transposons was generated using a pheatmap library and complete linkage hierarchical clustering.

Accession numbers: RNA-seq data reported in this study have been submitted to the GEO database and can be accessed online (see “Data availability” section).

### Immunofluorescence

Adult hermaphrodites raised at 20 °C or 25 °C were dissected in M9 buffer and flash frozen on dry ice before fixation for 1 min in methanol at −20 °C. After washing in PBS supplemented with 0.1% Tween-20 (PBST), primary antibody diluted in PBST was used to immunostaining overnight at 4 °C in a humid chamber. Primaries used were 1:50 OIC1D4 (Developmental Studies Hybridoma Bank). Secondary antibody staining was performed by using a Cy3 donkey anti-mouse or Cy-5 donkey anti-rabbit (Jackson Laboratories) overnight at 4 °C. For multiple antibody staining, the same protocol was used with minor changes. Gonads were fixed for 20 min in acetone at −20 °C^26^. GLH-1 rabbit antibody (1:200 dilution), PGL-3 rat antibody (1:200 dilution) (both antibodies were a kind gift from Karen Bennett), and OIC1D4 (Developmental Studies Hybridoma Bank) (1:50) were used for immunostaining overnight at 4 °C. Secondary antibodies used were donkey anti-rabbit Cy5, goat anti-rat Cy2, and donkey anti-mouse FITC (all Jackson Laboratories). Images were obtained using a LSM 710 laser scanning confocal and were taken using same settings as control samples or using an Axio Imager M2 Microscope (Zeiss) captured with an ORCA-Flash 4.0 digital camera (Hamamatsu) and Zen software (Zeiss). Images processed using ImageJ (version 2.1.0 and older). Marked P granule dysfunction was scored by eye as a loss of greater or less than 20% loss of P granules.

### DNA damage assay

Worms that were close to sterility were isolated and defined as sterile if they did not have any offspring as day 3 adults at 20 °C or day 2 adults at 25 °C. Fertile siblings of sterile worms were defined as “close to sterility”. The presence of the DNA damage response was determined by using a phospho-specific antibody targeting the phosphorylated consensus target site of ATM and ATR kinases (pS/TQ) (Cell Signaling Technology). This antibody has only been shown to stain a DNA damage response in the germline and was used as previously described^38^. Briefly, to stain isolated gonads, hermaphrodites were transferred to a poly-l-lysine-coated slide in M9. Extruded germlines were incubated with 2% para-formaldehyde for 10 min and permeabilized in PBT (PBS, 0.1% Triton-X) for 5 min. Primary antibody was diluted 1:500 in PBSB (PBS, 1% BSA) and incubated overnight at 4 °C. After three washes in PBSB, worms were incubated with secondary antibody donkey anti rabbit cy5 (Jackson Laboratories) overnight at 4 °C or 4 h at room temperature and nuclei were counterstained with DAPI (0.2 mg/mL) before mounting on a slide with Vectashield (Vector Laboratories). Images were taken using a Nikon Eclipse E800 microscope using NIS Elements software. Image analysis was performed in ImageJ (version 2.1.0 and older) by determining the ratio of pS/TQ positive germline cells to all germline cells.

### Gene editing by CRISPR

mCherry-tagged *znfx-1* was generated by CRISPR as previously described^77,78^. In brief, a pre-assembled Cas9 ribonucleoprotein complex was injected with an mcherry insert containing homology overhangs with SP9 5ʹ modifications for efficient homology repair. The sequence of primers and gRNA can be found in Table S5. The vector expressing *rol-6(su1006)*, a dominant allele conferring a roller phenotype, was used as a co-injection marker; mcherry-positive animals were identified among the progeny of F1 rollers that segregated F2 twitchers.

### RNA FISH experiment

We used smFISH probes from Stellaris (Cer3-CAL Fluor® Red 610 Dye and Cer8-CAL Fluor® Red 610 Dye^52^, Mirage1-CAL Fluor® Red 610 Dye^18^). Wild type, *nrde-2(gg95)*, and *hrde-1(tm1200)* were grown at 25 °C for two generations. Mixed stage animals were washed off from nonstarved plates into microcentrifuge tubes. Wash once in M9 buffer and three times in 1 mL of 1× DEPC-treated PBS. Fixation was performed by suspending worms in 1 mL of fixation buffer [3.7% (vol/vol) formaldehyde in 1× DEPC-treated PBS] for 45 min rotating at room temperature. Fixed animals were washed twice in 1 mL of 1× DEPC-treated PBS and permeabilized overnight at 4 °C in 70% ethanol in DEPC-treated H_2_O.

The next day animals were washed once in 1 mL of wash buffer [10% (vol/vol) formamide in 2× RNase-free SSC] and hybridized in 100 μL of hybridization buffer (1 g dextran sulfate, 10 mg *E. coli* tRNA, 100 µL 200 mM vanadyl ribonucleoside complex (New England Biolabs, Inc.), 40 µL 50 mg/mL RNase free BSA (Ambion), 1 mL Formamide (deionized, Ambion), nuclease free water(Ambion) to 10 mL final volume) with 1.25 μM probe. Worms were incubated at 30 °C overnight, then, washed in 1 mL of wash buffer for 30 min at room temperature, and finally washed again in 1 mL wash buffer with 25 ng/mL DAPI counterstain for 30 min at room temperature. Animals were mounted for imaging on glass slides by using VECTASHIELD mounting media (Vector Laboratories) before confocal microscopy.

Images were taken with a Leica SP8 confocal microscope and image processing was done in ImageJ (version 2.1.0 and older).

### Reporting summary

Further information on research design is available in the Nature Research Reporting Summary linked to this article.

## Supplementary information

Reporting Summary

Supplementary information

## Data Availability

The following publicly available RNA-seq datasets were downloaded from the Gene Expression Omnibus (GEO, https://www.ncbi.nlm.nih.gov/geo/): GSE92690 (P granule RNAi experiment) and GSE87524 (*prg-1* experiment). The RNA-seq datasets for nrde data were uploaded to the GEO. GEO accession number for the nrde RNA-seq data is GSE116367. These RNA-seq datasets were used to generate Fig. 5a as well Fig. S4 and Table S4. The data supporting the findings of this study are available from the corresponding authors upon reasonable request. Source data are provided with this paper.

## Source data

Source data

## Code availability

Custom codes for RNA-seq analysis have been developed for this manuscript and can be accessed by using the following link: https://github.com/sfrenk/rna-seq_pipelines.
