# Supplementary material for: Germ granule dysfunction is a hallmark and mirror of Piwi mutant sterility
Source: Nat Commun. 2021 Mar 3;12:1420. doi: 10.1038/s41467-021-21635-0 (PMC7930041; doi:10.1038/s41467-021-21635-0)
Supplement: Supplementary file 1 — Reporting Summary [file 41467_2021_21635_MOESM1_ESM.pdf]

nature research

Corresponding author(s): Shawn Ahmed

Last updated by author(s): 2021 01 20

Reporting Summary

Nature Research wishes to improve the reproducibility of the work that we publish. This form provides structure for consistency and transparency in reporting. For further information on Nature Research policies, see our Editorial Policies and the Editorial Policy Checklist.

Statistics

For all statistical analyses, confirm that the following items are present in the figure legend, table legend, main text, or Methods section.

n/a	Confirmed	
☐	☒	The exact sample size (*n*) for each experimental group/condition, given as a discrete number and unit of measurement
☐	☒	A statement on whether measurements were taken from distinct samples or whether the same sample was measured repeatedly
☐	☒	The statistical test(s) used AND whether they are one- or two-sided<br>*Only common tests should be described solely by name; describe more complex techniques in the Methods section.*
☒	☐	A description of all covariates tested
☐	☒	A description of any assumptions or corrections, such as tests of normality and adjustment for multiple comparisons
☐	☒	A full description of the statistical parameters including central tendency (e.g. means) or other basic estimates (e.g. regression coefficient) AND variation (e.g. standard deviation) or associated estimates of uncertainty (e.g. confidence intervals)
☐	☒	For null hypothesis testing, the test statistic (e.g. *F*, *t*, *r*) with confidence intervals, effect sizes, degrees of freedom and *P* value noted<br>*Give P values as exact values whenever suitable.*
☒	☐	For Bayesian analysis, information on the choice of priors and Markov chain Monte Carlo settings
☒	☐	For hierarchical and complex designs, identification of the appropriate level for tests and full reporting of outcomes
☒	☐	Estimates of effect sizes (e.g. Cohen's *d*, Pearson's *r*), indicating how they were calculated

Our web collection on statistics for biologists contains articles on many of the points above.

Software and code

Policy information about availability of computer code

Data collection

Library preparation and sequencing was performed at the UNC School of Medicine High-Throughput Sequencing Facility (HTSF). Libraries were prepared from ribosome-depleted RNA and sequenced on an Illumina Hiseq 2500.
The following publicly available RNA-seq datasets were download from the Gene Expression Omnibus (https://www.ncbi.nlm.nih.gov/ geo/): GSE92690 (P granule RNAi experiment) and GSE87524 (prg-1 experiment).
Fluorescent images were analyzed in ImageJ (version 2.1.0 and older).

Data analysis

Adapter trimming was performed as required using the bbduk.sh script from the BBmap suite (version 37.36) (Bushnell) and custom scripts. Reads were then mapped to the C. elegans genome (WS251) using hisat2 (version 2.1.0; Kim et al., 2013) with default settings and read counts were assigned to protein-coding genes using the featureCounts utility from the Subread package (version 1.5.3; Liao et al., 2014) . For multimapping reads, each mapping locus was assigned a count of 1/n where n=number of hits. Differentially expressed genes were identified using DESeq2 (version 1.16.1), and were defined as changing at least 2-fold with FDR-corrected p-value < 0.01. For analysis of transposon RNAs, reads were mapped to the C. elegans transposon consensus sequences downloaded from Repbase (http://www.girinst.org/repbase/) with bowtie (version 1.2.2; Langmead et al., 2009) using the options -M 1-v 2. Transposons with fewer than 10 counts in each sample were excluded from further analysis. Counts were normalized to the total number of mapped reads for each library for the prg-1 dataset, or to the total number of non-ribosomal mapped reads for all other datasets. A pseudocount of 1 was added to each value to avoid division by zero errors. Analysis of sequencing data and plot creation was performed using the R statistical computing environment (R Core Team, 2013). The custom codes for RNA-seq analysis have been developed for this manuscript and can be accessed by using the following link: https:// github.com/sfrenk/rna-seq_pipelines.

For manuscripts utilizing custom algorithms or software that are central to the research but not yet described in published literature, software must be made available to editors and reviewers. We strongly encourage code deposition in a community repository (e.g. GitHub). See the Nature Research guidelines for submitting code & software for further information.

Data

Policy information about availability of data

All manuscripts must include a data availability statement. This statement should provide the following information, where applicable:

- Accession codes, unique identifiers, or web links for publicly available datasets
- A list of figures that have associated raw data
- A description of any restrictions on data availability

The following publicly available RNA-seq datasets were downloaded from the Gene Expression Omnibus (GEO, https://www.ncbi.nlm.nih.gov/geo/): GSE92690 (P granule RNAi experiment) and GSE87524 (prg-1 experiment). The RNA-seq datasets for nrde data were uploaded to the GEO. GEO accession number for the nrde RNA-seq data is GSE116367. These RNA-seq datasets were used to generate figure 5a as well figure S4 and table S4. Source data are provided with this paper for figures 1b,c, 4a-d, i-j and S5a. All relevant data are available from the authors.

Field-specific reporting

Please select the one below that is the best fit for your research. If you are not sure, read the appropriate sections before making your selection.

☒ Life sciences ☐ Behavioural & social sciences ☐ Ecological, evolutionary & environmental sciences

For a reference copy of the document with all sections, see nature.com/documents/nr-reporting-summary-flat.pdf

Life sciences study design

All studies must disclose on these points even when the disclosure is negative.

Sample size	Samples sizes are noted in Figure legends. No statistical methods were used to predetermine sample size. Comparable sample sizes were chosen that allowed data reproducibility for each experimental conditions. For all data, positive and negative controls were included whenever possible. The samples sizes provided for each experiment are typical of those used in the field.
Data exclusions	No data exclusions.
Replication	All experimental conditions could be reproduced with success and specific reproducibility statements are added to each figure legend where appropriate.
Randomization	Experimental groups were based on similar condition (age of the animal, mutant type, temperature, generation). Some experiments (IFs and RNAi experiments were performed independently by more than one author of the paper).
Blinding	We were not blinded to group allocation during experiments and outcome assessment. However, control vs. experimental samples were always treated equally and in parallel.

Reporting for specific materials, systems and methods

We require information from authors about some types of materials, experimental systems and methods used in many studies. Here, indicate whether each material, system or method listed is relevant to your study. If you are not sure if a list item applies to your research, read the appropriate section before selecting a response.

Materials & experimental systems

n/a	Involved in the study	
☐	☒	Antibodies
☒	☐	Eukaryotic cell lines
☒	☐	Palaeontology and archaeology
☐	☒	Animals and other organisms
☒	☐	Human research participants
☒	☐	Clinical data
☒	☐	Dual use research of concern

Methods

n/a	Involved in the study	
☒	☐	ChIP-seq
☒	☐	Flow cytometry
☒	☐	MRI-based neuroimaging

Antibodies

Antibodies used	Primary antibodies: OIC1D4; Developmental Studies Hybridoma Bank; Phospho-ATM/ATR Substrate Motif [(pS/pT) QG] (Cell Signaling Technology), GLH-1 antibody, PGL-3 antibody. Secondary antibodies: Cy3 donkey anti-mouse, Cy-5 donkey anti-rabbit, donkey anti-rabbit Cy5, goat anti-rat Cy2and donkey anti-mouse FITC were all supplied by Jackson Laboratories.
Validation	References on manufacturer's website:<br>OIC1D4:

Asymmetric movements of cytoplasmic components in Caenorhabditis elegans zygotes.
Strome S.
Journal of embryology and experimental morphology 97 Suppl. (1986 Oct): 15-29.
Characterization of a germ-line proliferation mutation in C. elegans. Strome S.
Development (Cambridge, England) 116.3 (1992 Nov): 755-66.
Identification of immunological reagents for use in the study of freshwater planarians by means of whole-mount immunofluorescence and confocal microscopy.
Sanchez Alvarado A.
Genesis (New York, N.Y. : 2000) 32.4 (2002 Apr): 293-8.
Maternal mRNAs are regulated by diverse P body-related mRNP granules during early Caenorhabditis elegans development. Evans TC.
The Journal of cell biology 182.3 (2008 Aug 11): 559-72.
Linker histone HIS-24 (Hl.1) cytoplasmic retention promotes germ line development and influences histone H3 methylation in Caenorhabditis elegans.
Schulze E.
Molecular and cellular biology 27.6 (2007 Mar): 2229-39.
pS/TQ:
Sun, K., Tang, S., et al. (2019), 'Oxidized ATM-mediated glycolysis enhancement in breast cancer-associated fibroblasts contributes to tumor invasion through lactate as metabolic coupling.', EBioMedicine, 41, pp. 370-383
Sato-Carlton, A., Nakamura-Tabuchi, C., et al. (2018), 'Phosphorylation of the synaptonemal complex protein SYP-1 promotes meiotic chromosome segregation.', J Cell Biol, 217 (2), pp. 555-570
Possemato, A. P., Paulo, J. A., et al. (2017), 'Multiplexed Phosphoproteomic Profiling Using Titanium Dioxide and Immunoaffinity Enrichments Reveals Complementary Phosphorylation Events.', J Proteome Res, 16 (4), pp. 1506-1514 Genetics 208.2 (2018 Feb): 549-563.
GLH-1 antibody validation: Gruidl et al., 1996;
PGL-3 antibody validation: Kawasaki et al., 2004.

Animals and other organisms

Policy information about studies involving animals; ARRIVE guidelines recommended for reporting animal research

Laboratory animals	Caenorhabditis elegans, N2 strain (Bristol) or derived from this wild type strain. Animals of different ages were used throughout this study and the age of the animals for specific experiments is provided in the manuscript.
Wild animals	No wild animals were used in this study.
Field-collected samples	No field collected samples were used in this study.
Ethics oversight	No ethnical approval is required to work with Caenorhabditis elegans.

Note that full information on the approval of the study protocol must also be provided in the manuscript.
