# Supplementary material for: Germ granule dysfunction is a hallmark and mirror of Piwi mutant sterility
Source: Nat Commun. 2021 Mar 3;12:1420. doi: 10.1038/s41467-021-21635-0 (PMC7930041; doi:10.1038/s41467-021-21635-0)
Supplement: Supplementary file 2 — Supplementary information [file 41467_2021_21635_MOESM2_ESM.pdf]

Supplemental Information for: Germ granule dysfunction is a hallmark and mirror of Piwi mutant sterility

Supplementary Table 1

	fertile	sterile
	20˚C	
wt	0.00% (n=12)	NA
prg-1(n4357)	36.36% (n=11)	100.00% (n=5)
prg-1(tm872)	87.50% (n=8)	92.31% (n=13)
nrde-4(gg131)	33.33% (n=6)	90.91% (n=11)
nrde-1(yp4)	20.00% (n=5)	87.50% (n=8)
	25˚C	
wt	0.00% (n=35)	NA
mut-14(pk738)	NA	85% (n=15)
rsd-6(yp11)	0.00% (n=10)	100.00% (n=32)
nrde-2(gg95)	0.00% (n=10)	100.00% (n=25)
rbr-2(tm1231)	0.00% (n=10)	100.00% (n=32)
hrde-1(tm1200)	0.00% (n=10)	100.00% (n=31)

Table 1: Quantification of P granule defects in the germline. Sterile Piwi pathway genome silencing mutants were stained with P granule antibody. Germlines arms were semi-qualitatively scored for P granules. If 20% or more of the germline arm exhibited P granules abnormalities (complete or partial loss of P granules) germlines were scored as abnormal.

Supplementary Table 2

	P granule loss
20˚C	
wt L4	0.00% n=5
wt day 1	0.00% n=5
prg-1(n4357) L4	28.57% n=7
prg-1(n4357) day 1	50.00% n=10
nrde-4(gg131) L4	60.00% n=5
nrde-4(gg131) day 1	50.00% n=4
25˚C	
hpl-2(ok1061) L4	55.56% n=9
hpl-2(ok1061) day 2 fertile	50.00% n=6
hpl-2(ok1061) day 2 sterile	100% n=3
hpl-2(tm1489) L4	37.50% n=8
hpl-2(tm1489) day 2 fertile	16.67% n=6
hpl-2(tm1489) day 2 sterile	85.71% n=7

Table 2: Quantification of P granule defects in the developing germline. Late generation Piwi pathway genome silencing mutants were stained with P granule antibody at the indicated developmental stage. Germline arms were semi-qualitatively scored for P granules. If 20% or more of the germline arm exhibited P granules abnormalities (complete or partial loss of P granules) germlines were scored as abnormal.

Supplementary Figure 1

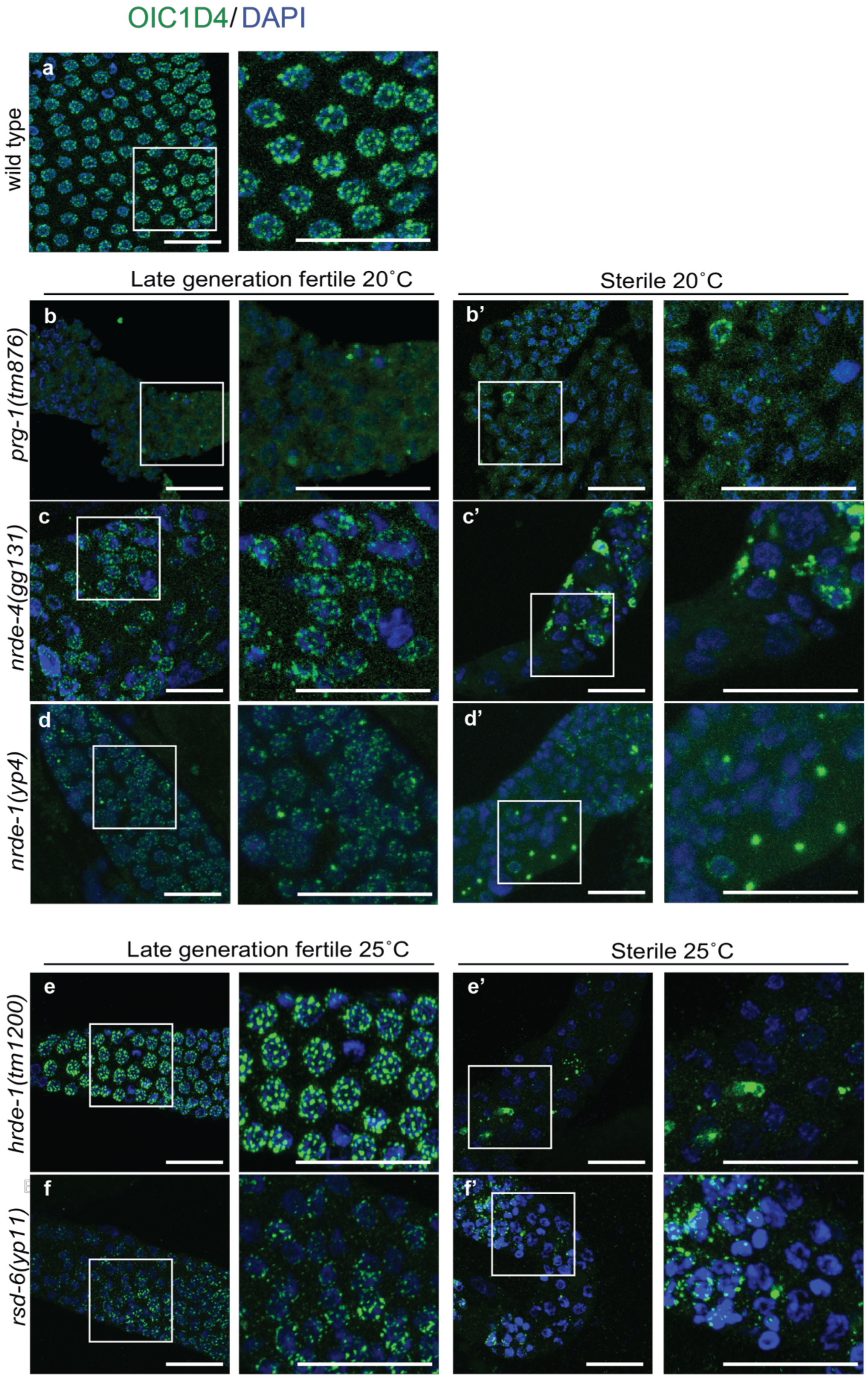

Fig. S1: Related to Figure 2. P granule defects in *prg-1, nrde-4, hrde-1, nrde-1, hrde-1 and rde-6*. Germlines of sterile Day 2-3 adult animals were stained using the OIC1D4 antibody against P granules (green) and DAPI (blue). More than 5 independent OIC1D4 antibody immunofluorescence experiments were performed with similar results on wild type and different mutants and at least 5 animals were scored in each category (Table S1). Representative images are shown. Scale bars indicate 20µm. **a** Control animals contain uniform puncta of P granule staining surrounding each nucleus. **b-f** Late generation fertile *prg-1(tm876), nrde-4(gg131), nrde-1(yp4)*, *hrde-1(tm1200)* and *rsd-6(yp11)* and animals displayed P granule staining similar to wild type except for *prg-1(tm876).* **b'-f'** Sterile *prg-1(tm876), nrde-4(gg131), nrde-1(yp4)*, *hrde-1(tm1200)* and *rsd-6(yp11)* with P granule abnormalities.

Supplementary Figure 2

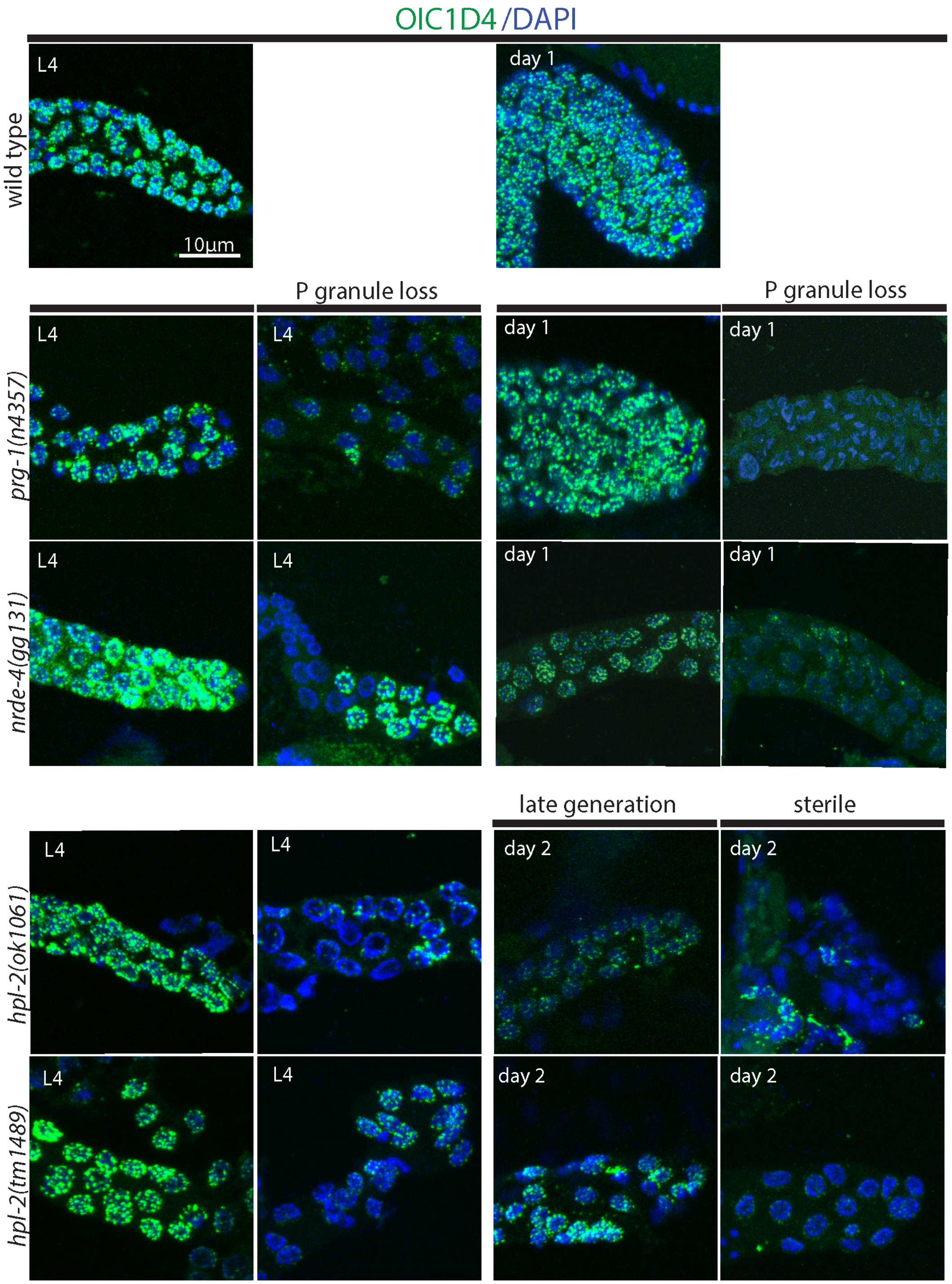

Fig. S2: P granule defects are observed in the early stages of germline development. *prg-1* (20˚C), *nrde-4* (20˚C) and *hpl-2* (25˚C) mutants were stained with the OIC1D4 antibody against P granules (green) and DAPI (blue) at L4 stage and day 1/day 2 of adulthood as indicated from a late generation population of worms. Defects at L4 stage and day 1/day 2 of adulthood could be observed for all analyzed mutants indicating that the defects can already appear in young germlines. P granule defects were predominant in sterile day 2 *hpl-2* mutants compared to animals that were still fertile. Reproducibility was obtained by imaging P granules in L4 and day 1 wild type and Piwi mutants in at least 3 independent experiments with similar results. Animals in each category were scored for P granule defects in Table S2.

Supplementary Table 3

	Total Worms Scored	% Fertile	% Sterile
pgl-1 F1 at 25˚C	91	22%	78%
pgl-1 F2 at 25˚C	71	0%	100%

Table 3: Related to Figure 4. Fertility of *pgl-1* mutants at 25°C. 20˚C P0 *pgl-1* mutants were shifted to 25˚C and fertility was measured for the F1 generation. Fertile F1 worms were cloned and the fertility of the F2 generation was measured.

Supplementary Figure 3

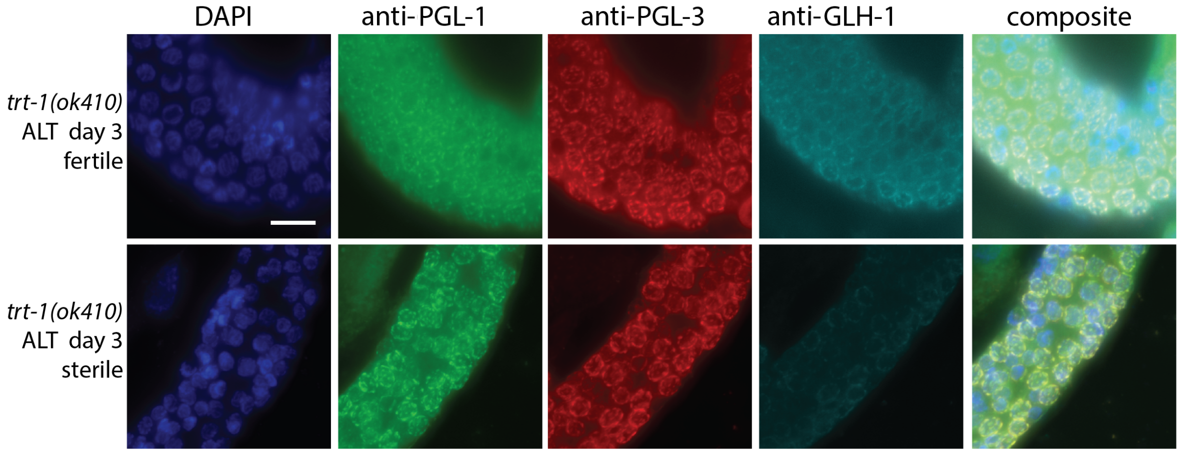

Fig. S3: P granules in sterile and late generation ALT strains. *trt-1* ALT strains were grown for more than 200 generations and worms became sterile when singled for a few generations from crowded plates. Late generation fertile and sterile animals were stained with antibodies against PGL-1, PGL-3 and GLH-1. (*trt-1(ok410)* ALT: fertile n=11; sterile n=8). Reproducibility was confirmed by obtaining similar results for two different *trt-1(ok410)* ALT strains. Representative images are shown. Scale bar indicates 10μm.

Supplementary Table 4

**gene**	***prg-1***	***nrde-1***	***nrde-4***
C06B3.7	4.331128036	1.732445354	2.669978253
dhs-26	3.88587979	3.752818032	1.793937273
bath-45	3.604851731	5.5002102	2.319444032
fbxb-97	3.555301139	3.002992933	3.278539846
irg-2	3.519633623	2.605415087	2.189754742
pud-4	3.226292885	2.956152303	4.407697185
pud-3	3.097392415	3.143142026	4.328678969
R09E10.2	2.745900789	5.100997358	4.251177196
Y53F4B.5	2.636725232	5.821227047	4.162462556
K08D12.6	2.631068263	2.955437046	0.277803871
R03H10.6	2.469258519	4.82098578	3.295830487
Y58A7A.5	2.397260966	1.795895	2.143861408
pud-1.1	2.299685196	2.556376909	3.17781014
T22B7.7	2.276777478	3.01143186	2.207919501
fbxa-224	2.248998131	4.226992478	1.979961678
swt-6	2.144598564	3.153313277	0.277029378
pud-2.1	2.119890425	2.781615959	2.880747229
pud-2.2	2.094123631	2.779337879	2.796566876
pud-1.2	2.080250623	2.559790088	3.000944771
catp-3	2.014970724	2.436698711	2.674464594

Table 4: Related to Figure 5. Genes upregulated in late-generation *prg-1* mutants and in sterile *nrde-1* and *nrde-4* mutant L4 larvae.

Supplementary Figure 4

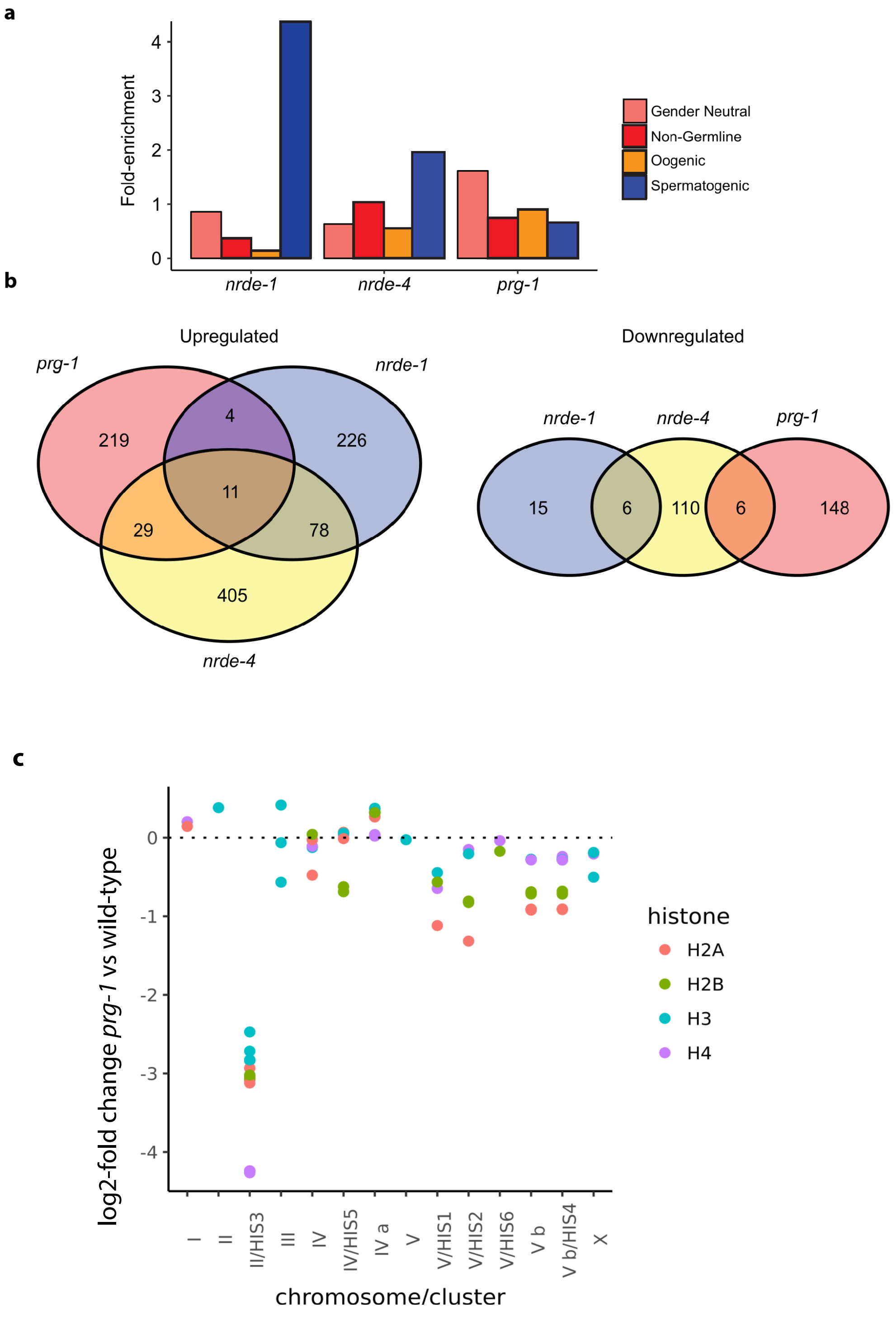

Fig. S4: Transcriptional analysis of *nrde* mutants. a Enrichment of germline-expressed genes in genes upregulated in *nrde-1* and *nrde-4* mutants. **b** Number of genes up and downregulated in *nrde-1* and *nrde-4* mutants. **c** Histone genes are down regulated in late generation *prg-1*.

Supplementary Figure 5

a

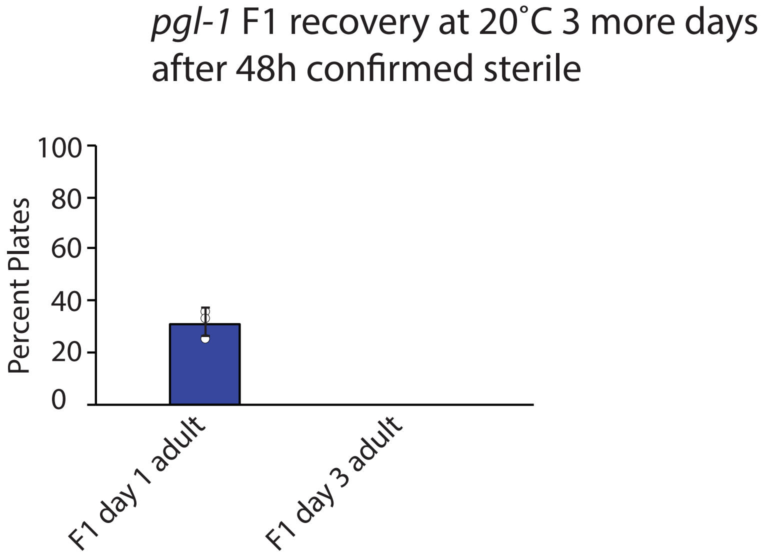

b

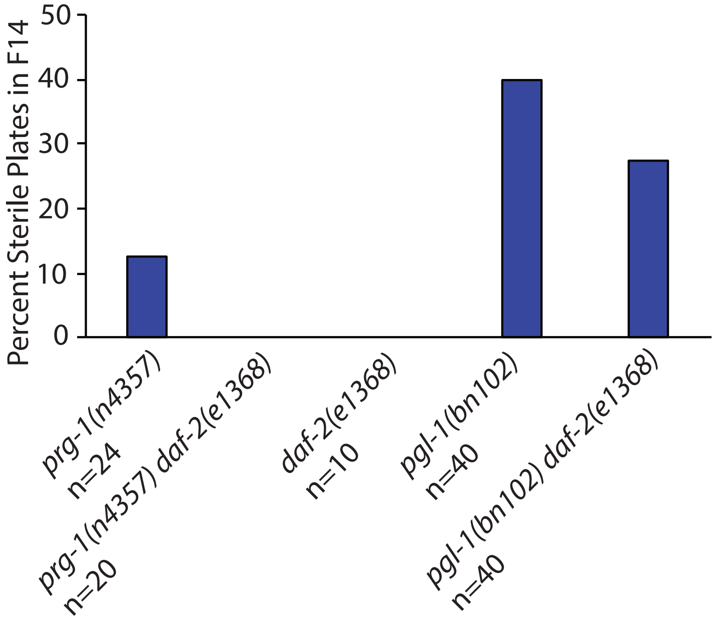

Fig. S5: *pgl-1* recovery and rescue. a 25˚C F1 *pgl-1* mutant recovery for animals that were still sterile 48h post 25˚C incubation as day 3 adults. Recovery of fertility was observed during day 3 and 6 of adulthood for a small population of animals. Individual data points from 3 independent experiments are shown in white circles. Error bars represent the standard deviation. (A schematic cartoon summarizing experimental conditions is shown in figure S6b and d) **b** Percent sterility of *prg-1, pgl-1, daf-2* and double mutants at generation F14. *prg-1 daf-2* does not show any sterility at this generation, while *pgl-1 daf-2* has a similar percentage of sterility as the *pgl-1* single mutant indicating that *pgl-1* sterility cannot be rescued by *daf-2* mutations.

Supplementary Figure 6

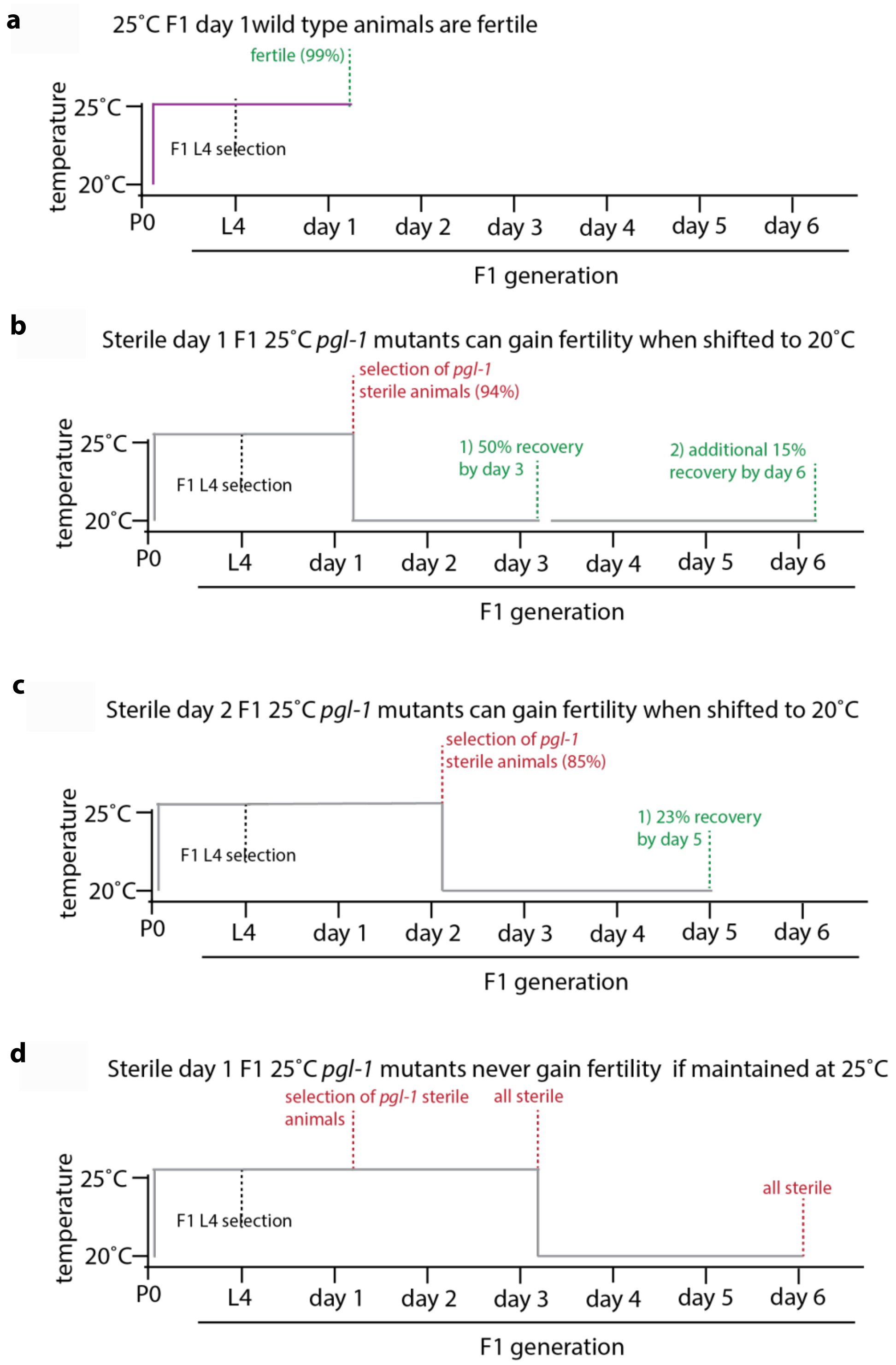

Fig. S6: Schematic summary for time course for *pgl-1* F1 recovery from 25˚C related to Figure 4i, 4j, S4a. a Purple line indicates wild type animal experimental temperature over the developmental time course. 25˚C day 1 wild type animals are fertile. **b**,**c and d**: Grey lines indicate experimental conditions for *pgl-1* animals at their specific temperature over the developmental time course. Sterile 25˚C F1 day 1 and day 2 *pgl-1* adults can develop fertility when shifted to 20˚C (b, c) but will remain sterile when maintained at 25˚C until day 3 of adulthood or longer (d).

Supplementary Table 5

Description	Primer name	Primer sequence
pgl-1(bn101) fw primer	MS_bn101_f93	TGGATGATGTGATTGCCGAGGAAC
pgl-1(bn101) rv primer	MS_bn101_r94	GTCAACGCATTTGATAGCTGCAAGC
pgl-1(bn102) fw primer	MS_bn102_fw_97	ACCGCATCATTGTTTAGTGGACTC
pgl-1(bn101) rv primer	MS_bn102_rv_98	GAGCGGAAGTCTTTCCAGGATTG
prg-1(n4357) fw primer	n43_fw	CAACACCCTATCGACAGATCG
prg-1(n4357) rv primer	n43_rv	CCTTGGACTTTCCGGCC
Teg PCR donor *znfx-1* fw	DD155 TEG	/5Sp9/cttgtttcagACCAATTCGCCAACCGTATTCAATGGTCTCAAAGGGTGAAGAAGA
Teg PCR donor *znfx-1* rv	DD157 TEG	/5Sp9/GGCGGCGGGAGCCCTGGGGGGCGAGGTTTCTGACcttatacaattcatccatgc

Description	guide RNA name	guide RNA sequence
znfx-1 crRNA	DD_RNA_24 crRNA	ATG AGGTTTCTGACCATTGAATA
